# Oseltamivir Population Pharmacokinetics in the Ferret: Model Application for Pharmacokinetic/Pharmacodynamic Study Design

**DOI:** 10.1371/journal.pone.0138069

**Published:** 2015-10-13

**Authors:** Micaela B. Reddy, Kuo-Hsiung Yang, Gauri Rao, Craig R. Rayner, Jing Nie, Chandrasena Pamulapati, Bindumadhav M. Marathe, Alan Forrest, Elena A. Govorkova

**Affiliations:** 1 Department of Drug Metabolism and Pharmacokinetics, Hoffmann-La Roche Inc., Nutley, New Jersey, United States of America; 2 Department of Pharmacy Practice, University of Buffalo, Buffalo, New York, United States of America; 3 Department of Infectious Diseases, St. Jude Children's Research Hospital, Memphis, Tennessee, United States of America; Mount Sinai School of Medicine, UNITED STATES

## Abstract

The ferret is a suitable small animal model for preclinical evaluation of efficacy of antiviral drugs against various influenza strains, including highly pathogenic H5N1 viruses. Rigorous pharmacokinetics/pharmacodynamics (PK/PD) assessment of ferret data has not been conducted, perhaps due to insufficient information on oseltamivir PK. Here, based on PK data from several studies on both uninfected and influenza-infected groups (i.e., with influenza A viruses of H5N1 and H3N2 subtypes and an influenza B virus) and several types of anesthesia we developed a population PK model for the active compound oseltamivir carboxylate (OC) in the ferret. The ferret OC population PK model incorporated delayed first-order input, two-compartment distribution, and first-order elimination to successfully describe OC PK. Influenza infection did not affect model parameters, but anesthesia did. The conclusion that OC PK was not influenced by influenza infection must be viewed with caution because the influenza infections in the studies included here resulted in mild clinical symptoms in terms of temperature, body weight, and activity scores. Monte Carlo simulations were used to determine that administration of a 5.08 mg/kg dose of oseltamivir phosphate to ferret every 12 h for 5 days results in the same median OC area under the plasma concentration-time curve 0–12 h (i.e., 3220 mg h/mL) as that observed in humans during steady state at the approved dose of 75 mg twice daily for 5 days. Modeling indicated that PK variability for OC in the ferret model is high, and can be affected by anesthesia. Therefore, for proper interpretation of PK/PD data, sparse PK sampling to allow the OC PK determination in individual animals is important. Another consideration in appropriate design of PK/PD studies is achieving an influenza infection with pronounced clinical symptoms and efficient virus replication, which will allow adequate evaluation of drug effects.

## Introduction

Seasonal and pandemic influenza are important public health concerns. Pandemics such as the 2009 influenza A (H1N1) virus pandemic can stress hospital resources, including emergency departments and intensive care units [1], while causing fever, respiratory symptoms, weakness, and myalgias in patients, and potentially causing critical illness and death [2]. While vaccination is effective at preventing influenza infection, epidemics still occur annually. Specific influenza antiviral drugs, such as the neuraminidase inhibitors oseltamivir and zanamivir, are recommended both in preventative use and in treatment of infected patients [3,4,5]. Oseltamivir (Tamiflu^®^, F. Hoffmann-La Roche Ltd.) is an orally administered antiviral drug that is approved for the treatment of influenza A and B in adults and children (including full term neonates) who present with symptoms typical of influenza when influenza virus is circulating in the community, and for the prophylaxis of influenza in patients aged 1 year or older [6, 7]. These approvals are based on extensive efficacy and safety data obtained from clinical trials of oseltamivir across a wide range of patient groups [8–15]. Oseltamivir has been used worldwide with more than 65 million treatment courses administered to pediatric, adult, and elderly patients [16]. The ability to treat renally and hepatically impaired patients with oseltamivir and its low potential for drug-drug interactions are additional benefits for this medication [6,7,16].

Oseltamivir is a prodrug that is administered as a phosphate salt (oseltamivir phosphate; OP). It is then converted by hepatic carboxylesterases to the active metabolite oseltamivir carboxylate (OC). In humans, OP is readily absorbed and converted to OC, which is detectable in plasma within 30 min, and the absolute bioavailability for OC is 80%. Peak plasma concentrations of OC are attained in about 3–4 h, and the apparent half-life is 6–10 h, with elimination primarily through renal excretion of OC [16,17]. Clinical studies have established the safety and tolerability of oseltamivir, with no significant safety concerns in patient populations for which oseltamivir has been approved [18]. Safety and tolerability have been observed even at doses 6-fold higher than the standard treatment dose of 75 mg twice daily [19]. While much is known about OP and OC pharmacokinetics (PK) in humans, ferret PK has been less well characterized, with only limited information available in peer-reviewed literature [17,20]. Additionally, human PK models have been described for OP and OC [21,22,23].

Preclinical studies in animal models can provide desirable information about optimal drug regimens, particularly for highly pathogenic H5N1 influenza viruses for which clinical trials are not available. The ferret model is an excellent small animal model for studying influenza virus infection because the ferret is naturally susceptible to influenza, and the course of the illness is similar to that of humans. The ferret model was used to assess not only pathogenicity and transmissibility of influenza viruses [24,25,26,27] but also oseltamivir efficacy against lethal and non-lethal challenges with influenza viruses. It was demonstrated that oseltamivir administration decreased signs of infection in the ferret and impeded viral pneumonia development by reducing the spread of the pandemic H1N1pdm09 virus in the lungs [28]. The observed clinical outcome of infection in ferrets depends on the H5N1 strain and virus dose. For example, previous studies showed that inoculation of ferrets with as few as 10 EID_50_ (50% egg infectious doses) of A/Vietnam/1203/2004 (H5N1) virus caused systemic spread and death, whereas 10^6^ EID_50_ of A/Turkey/15/2006 (H5N1) virus were not lethal [29]. Prophylactic administration of oseltamivir has been shown to protect ferrets from influenza A (H3N2) virus infection, by reducing febrile and inflammatory responses [30], and to prevent morbidity and mortality from H5N1 influenza infection [31]. Elucidation of the PK of OP and its active metabolite OC in the ferret model can help with improved study design, clarify the translational implications of such studies, and enable PK/pharmacodynamic (PK/PD) assessment of factors driving efficacy.

Here, we present data for OP and OC PK from four studies in ferrets: (1) single and multiple doses of oseltamivir in both uninfected and influenza A (H5N1) virus infected ferrets, and multiple doses of oseltamivir in influenza A (H3N2) virus infected ferrets, with ketamine anesthesia prior to blood sample collection; (2) single and multiple doses of oseltamivir in both uninfected and influenza B virus infected ferrets with ketamine anesthesia prior to blood sample collection; (3) single and multiple doses of oseltamivir in uninfected healthy ferrets without anesthesia; and (4) single dose oseltamivir in healthy ferrets maintained under Saffan anesthesia. In previous modeling approaches, PK of both OP and OC were described with hepatic conversion of OP to OC [21,22]. Here we applied a simplified approach of a population PK model describing the PK of OC in the ferret after oral oseltamivir administration without explicitly including OP PK in the model. The model is used to determine whether covariates such as anesthesia and subclinical influenza virus infection have a significant impact on PK. The data and PK model are used as the basis for recommendations on appropriate PK/PD study design when using the ferret model to assess the efficacy of oseltamivir for a given strain of influenza virus.

## Materials and Methods

### Compounds

The active metabolite, OC ([3R,4R,5S]-4-acetamido-5-amino-3-[1-ethylpropoxy]-1-cyclohexene-1-carboxylic acid), the prodrug OP [ethyl(3R,4R,5S)-4-acetamido-5-amino-3-(1-ethylpropoxy)-1-cyclohexene-1-carboxylate], and the free base form of oseltamivir (OFB) were provided by F. Hoffmann-La Roche Ltd.

### PK Studies in a Ferret Model

Study 1 was approved by the Institute of Animal Use and Care Committee of the Institute of Laboratory Animal Science, Peking Union Medical College. Study 2 was approved by the St. Jude Animal Care and Use Committee and complied with the policies of the National Institutes of Health and the Animal Welfare Act. Studies 3 and 4 were approved by the UK Home Office and performed in accordance with the Animals (Scientific Procedures) Act of 1986.

Study 1 was conducted at the Institute of Laboratory Animal Science (ILAS) of the Chinese Academy of Medical Sciences (Beijing, China) in accordance with the Association for Assessment and Accreditation of Laboratory Animal Care (AAALAC International) guidelines and under the approval of the Institutional Animal Care and Use Committee (IACUC). The study included two parts. Part 1 was a study of PK in infected and uninfected animals, and Part 2 was a PK/PD study in infected animals that included sparse PK sampling. The influenza A/Hong Kong/433581/2009 (H3N2) and A/Shenzheng/406H/2006 (H5N1) strains were provided by Dr Honglin Chen (The University of Hong Kong, Pokfulam, Hong Kong, China).

In Part 1 of Study 1, the single dose part, young adult male ferrets (4–5 months old, 800–900 g) were inoculated with 10^2^ TCID_50_ (50% tissue culture infectious dose) of highly pathogenic A/Shenzheng/406H/2006 (H5N1) influenza virus and dosed with 5.0 or 12.5 mg/kg of OFB (corresponding to 6.56 or 16.4 mg/kg OP). Control uninfected ferrets were dosed at 5.0 mg/kg OFB. OFB was administered in a 50% sugar syrup in distilled water. Blood samples for PK analysis were collected at 0, 0.5, 1, 1.5, 2, 4, 6, 8, and 12 h after dosing. There were three control uninfected ferrets, four H5N1-infected ferrets in the low dose group, and three H5N1-infected ferrets in the high dose group, totaling ten in the study.

In Part 2 of Study 1, the multiple dose part, sparsely sampled animals were inoculated with either 10^2^ TCID_50_ of A/Shenzheng/406H/2006 (H5N1) or 10^6^ TCID_50_ of A/Hong Kong/433581/2009 (H3N2) influenza viruses. Animals were administered 0, 12.5, or 25.0 mg/kg of OFB (corresponding to 0, 16.4, and 32.9 mg/kg OP, respectively) every 12 h (q12h) for 5 days beginning 24 h after inoculation. Blood samples for PK analysis were collected at 1, 4, 49, and 52 h after the first dose. There were six ferrets in each group, totaling 36 for the study, but only 24 with PK data because 12 of the animals were not administered drug. Animals were anesthetized with ketamine (10 mg/kg) injected intramuscularly before blood sampling from the jugular vein and dosing. Animals were anesthetized with ketamine (10 mg/kg and domitor (0.1 mg/kg) prior to euthanasia. The virus doses were selected to cause symptoms of influenza while allowing a full time-course of disease progression data. Prior to bioanalysis, the virus was inactivated using irradiation. An IPTT-300 encapsulated microchip (Bio Medic Data Systems, United States of America) was implanted subcutaneously in each ferret one week before virus challenge. The body temperature of infected ferrets was recorded twice daily with a microchip reader (DAS-6000, Bio Medic Data Systems, United States of America). Body weight was estimated once daily with an electronic balance.

For Study 1, the activity scores combined respiratory symptoms and activity. A score of zero indicated no nasal symptoms and a fully playful ferret. A score of one indicated nasal rattling or sneezing and a ferret that responded to play overtures but that did not initiate play. A score of two indicated nasal discharge and a ferret that was alert but not playful. A score of three indicated that mouth breathing was necessary and the ferret was not playful or alert.

Study 2 was conducted at St. Jude Children's Research Hospital (Memphis, Tennessee, United States of America) under applicable laws and guidelines and after approval from the IACUC. The influenza B/Yamagata/16/1988 strain was provided by Dr Larisa Gubareva (Centers for Disease Control and Prevention, Atlanta, Georgia, United States of America). A control group of young adult male ferrets (3–5 months old, 600–900 g; Triple F Farms, Sayre, Pennsylvania, United States of America) were given a single oral dose of OP of 1.0 mg/kg on day 1, 5.0 mg/kg on day 3, and 25.0 mg/kg on day 5 (i.e., a single 0.76, 3.8, and 19 mg/kg dose of OFB), administered in a 50% sugar syrup in distilled water. There were three blood sample collection schemes for PK analysis (two ferrets per scheme). The first scheme was: 0.5, 4, 12 h after dosing on day 1; 2, 8, 12 h after dosing on day 3; and 1, 6, 12 h after dosing on day 5. The second scheme was: 2, 8, 12 h after dosing on day 1; 1, 6, 12 h after dosing on day 3; and 0.5, 4, 12 h after dosing on day 5. The third scheme was: 1, 6, 12 h after dosing on day 1; 0.5, 4, 12 h after dosing on day 3; and 2, 8, 12 h after dosing on day 5. Ferrets in the treatment group were inoculated with 10^7^ plaque forming units (PFU) of B/Yamagata/16/1988 influenza virus, and dosed orally at OP doses of 1.0, 5.0, or 25.0 mg/kg q12h for 5 days beginning 24 h after inoculation (six ferrets per dose). The PK sampling scheme was identical to PK control groups (two ferrets per scheme). Ketamine anesthesia (25.0 mg/kg) was used prior to blood sample collection from the jugular and sacrifice by intracardiac injection of Euthanasia V solution. The inoculation dose was selected to maximize chances of seeing symptoms for this relatively low pathogenic influenza strain.

Activity scores were recorded daily: a score of “0” was given to alert and playful animal, score of “1”–alert, but playful when stimulated, score of “2”–alert, but not playful when stimulated, score of “3”–neither alert nor playful when stimulated [32].

Study 3 was conducted at Retroscreen Ltd., Whitechapel (London, United Kingdom) under the approval of the IACUC. OP was administered in liquid cat food (Liquivite, Highgate, London, United Kingdom). Influenza virus challenge was not used in this study. Young adult female ferrets (794–919 g) were dosed orally at either 5.0 or 25.0 mg/kg of OP (four ferrets per dose), i.e., 3.8 or 19 mg/kg OFB. After a month-long washout, they were dosed at either 5.0 or 25.0 mg/kg of OP (four ferrets per dose) q12h for 5 days. Plasma sampling was sparse, with four samples per animal. Blood samples for PK analysis were taken from the tail vein at 0.5 and 2 h, or 1 and 4 h, as well as at 8 and 12 h after single dose drug administration and at the same times after the last dose on day 5 of repeat dosing.

Study 4 was conducted at Biodynamics Research Ltd., of Cardiff Medicentre, Heath Park (Cardiff, Wales, United Kingdom) under the approval of the IACUC. Influenza virus challenge was not used in this study. Young adult female ferrets (760–1280 g; Harlan UK Limited, Hillcrest Research Station, Dodgeford Lane, Belton, Loughborough, Leicestershire, United Kingdom) were administered a single 5.0 mg/kg dose of OFB (four ferrets per dose) in sterile water by gavage. Although ^14^C-radiolabeled compound was administered, plasma concentrations were determined using high-performance liquid chromatographic–double mass spectrometry (HPLC/MS/MS) as with the other studies. Blood samples for PK analysis were taken from the jugular vein at 0, 0.25, 0.5, 1, 2, 4, 8, 12, and 24 h. After dosing, the ferrets were maintained under Saffan anesthesia, a steroidal preparation containing two pregnanediones (alfaxalone and alfadolone acetate), for 12 h. At 12 h the effects of the anesthesia were reversed using Antisedan. Animals were euthanized by injecting 2.0 ml Saffan intramuscularly followed by 2.0 ml sodium pentabarbitone intracardially.

### OC Plasma Concentrations

Blood samples were collected into ethylenediaminetetraacetic acid (EDTA) tubes, cooled in ice, and the plasma harvested by centrifugation at 4°C. The plasma was frozen and stored at 20°C or less depending on the study. For all studies, OC concentrations in plasma samples were determined by a HPLC-MS/MS method described by Wiltshire et al. [33].

### Non-compartmental Analysis (NCA)

The PK concentrations of OP and OC in uninfected animals after a single oral administration of a 5.0 mg/kg dose of OP for all four studies described above were evaluated by NCA in Phoenix^®^WinNonlin^®^ (Pharsight Corp, St. Louis, Missouri, United States of America). Samples with concentrations of below the limit of quantitation (LOQ) were set to zero. The maximum observed plasma concentration (C_max_), the time to reach the maximum observed plasma concentration (T_max_), and the area under the plasma concentration-time curve (AUC) 0–12 h (AUC_0–12h_) were reported.

The PK parameters were calculated from plasma concentration data from individual animals, where possible, so that mean values and standard deviations could be reported. For Study 3, in which animals received no anesthesia, data were sparse, but PK was still well-characterized by PK sampling at only 0.5, 2, 8, and 12 h, or 1, 4, 8, and 12 h after dosing. Therefore NCA parameters were determined for individual subjects as was done for rich PK sampling. For Study 2 NCA of profiles in individual animals was not possible as there were only three PK samples in each animal per dose, and so the data were treated as composite data.

### OC PK Model

Data from Studies 1, 2, and 3 were used in PK model development. Data from Study 4, conducted with uninfected ferrets maintained under Saffan anesthesia, were determined to be different from other studies based on NCA (i.e., for Study 4 the T_max_ was 7 h for OC and OP, but in the other studies the T_max_ for OP was 1 h and the T_max_ for OC was 3–4 h) and so were left out of the analysis. PK data used for model development are available in the S1 Table.

**Fig 1:**
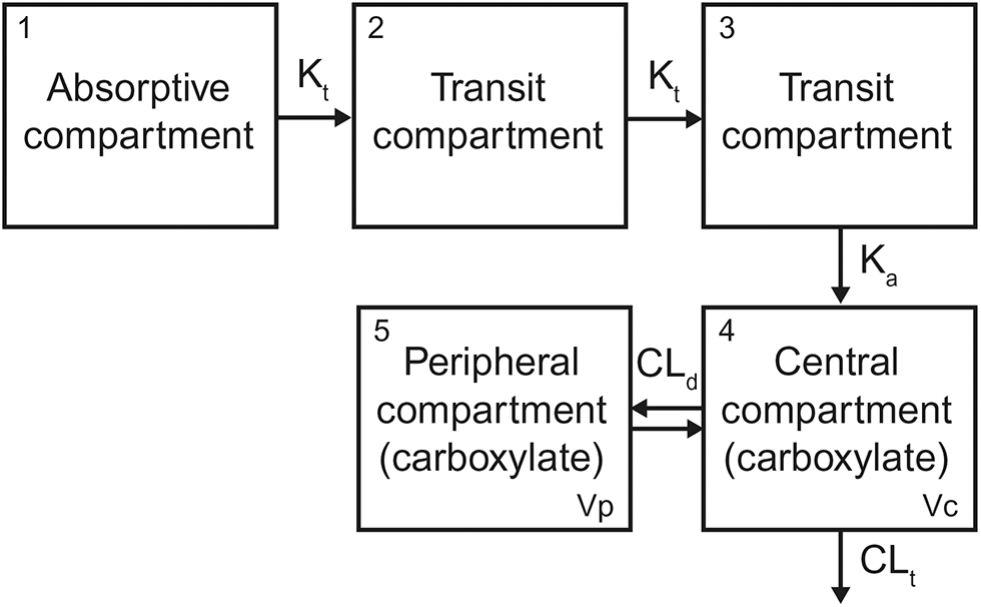
Schematic diagram of the oseltamivir PK model.

The structural model includes an oral absorption compartment with amount of OP (X_1_) that leads to two transit compartments to model the delay of OC appearance (Fig 1). The second transit compartment leads to a central and peripheral compartment for OC with amounts X_4_ and X_5_ and volumes V_c_ and Vp, respectively, with first-order clearance from the central compartment, CL_t_:

$$\frac{dX_{1}}{dt}=-K_{t}\times X_{1}$$ (1)

$$\frac{dX_{2}}{dt}=K_{t}\times X_{1}-K_{t}\times X_{2}$$ (2)

$$\frac{dX_{3}}{dt}=K_{t}\times X_{2}-K_{a}\times X_{3}$$ (3)

$$\frac{dX_{4}}{dt}=K_{a}\times X_{3}-\frac{CL_{d}\times X_{4}}{V_{c}}+\frac{CL_{d}\times X_{5}}{V_{p}}-\frac{CL_{t}\times X_{4}}{V_{c}}$$ (4)

$$\frac{dX_{5}}{dt}=\frac{CL_{d}\times X_{4}}{V_{c}}-\frac{CL_{d}\times X_{5}}{V_{p}}$$ (5)

X_2_ and X_3_ are the amount of OC in the first and second transit compartment, K_t_ is the first-order rate constant for transfer from one transit compartment to the next, K_a_ is the first-order absorption rate constant, and CL_d_ is the clearance into and out of the peripheral compartment. A two-compartment distribution model [34] was required to describe OC systemic PK. The parameter CL_t_ describes the elimination of OC without assuming a mechanism of elimination, and could include renal and/or metabolic elimination. The CL and V parameters were conditioned on two fractions: oral bioavailability (F) and fraction of parent changed to metabolite (Fm). The concentration of OC in the plasma is calculated as X_4_/V_c_. This empirical model structure was developed to describe the concentration of OC in the blood after OP administration. Only OC data, no OP data, were used in model parameterization. Although blood sampling was not done from the same physiological location for all studies (i.e., for Studies 1, 2, and 4, PK samples were drawn from the jugular vein, but for Study 3, PK samples were drawn from the tail vein), it was assumed that all PK samples were representative of the central compartment concentration.

To determine the appropriate structural model, OC PK data from individual ferrets in Study 1 (Part 1 ferrets with rich PK sampling) were fitted in an iterative two-stage approach (Adapt 5, Biomedical Simulations Resource, University of Southern California, Los Angeles, California, United States of America, bmsr.usc.edu). Several simpler model structures were explored before settling on the structural model in eqs (1)–(5). Model discrimination was by the Akaike information criterion (AIC). AIC, a measure of model goodness-of-fit, contains a penalty for the number of model parameters, which enables its use in selecting the model that provides the best balance between fit and complexity [35].

**Fig 2:**
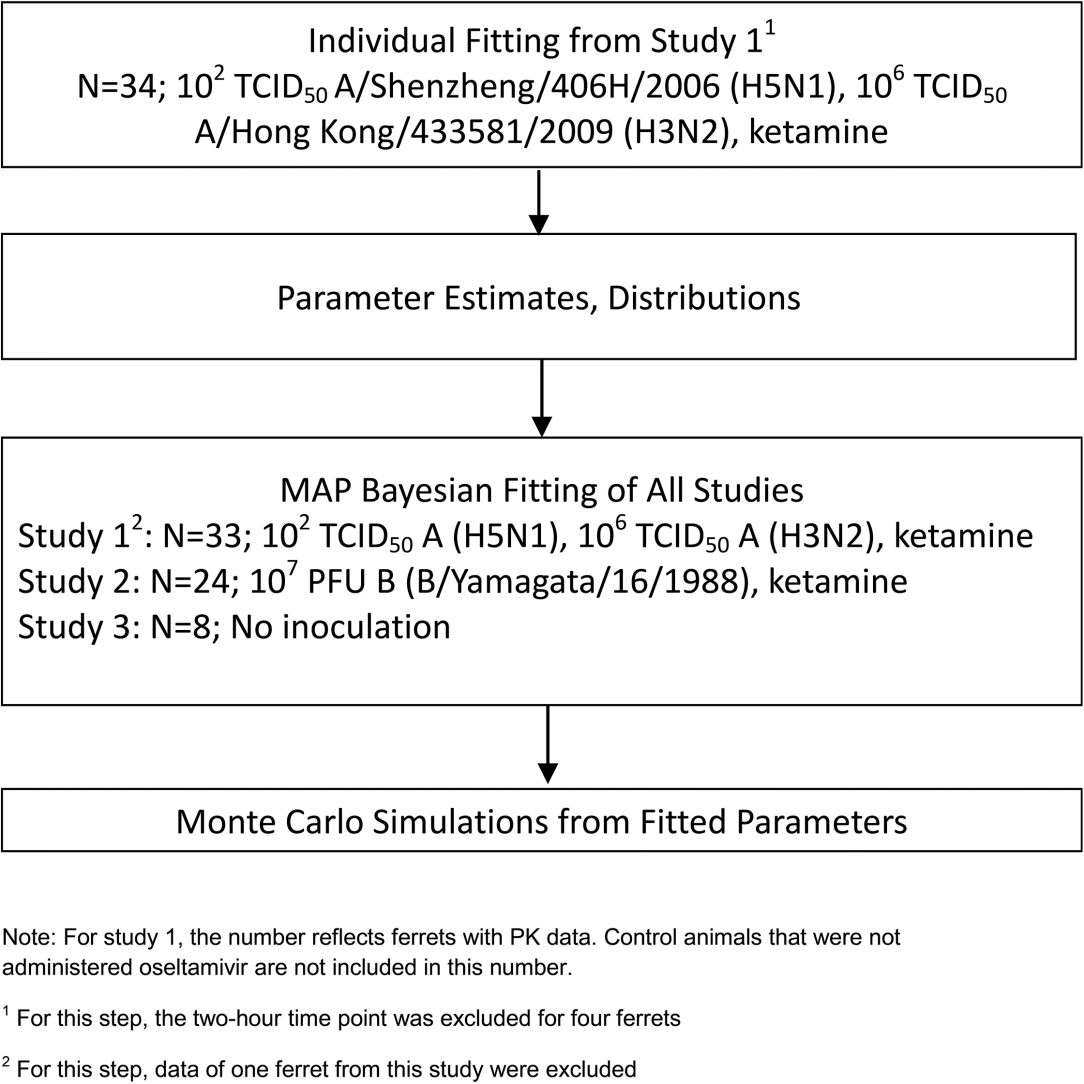
Flowchart of method used in model fitting.

During the first step of model parameterization (Fig 2), the goal of which was to ensure that good initial estimates of model parameters were obtained, OC PK data were first fit using maximum likelihood. The 2-h time points from Study 1 ferrets 1, 2, 7 and 8 were outlier data points and thus excluded from this analysis. In the second step, the resulting median and variance parameters were used as priors in the Bayesian maximum a posteriori probability (MAP) estimator [36] to refit the OC PK data from Studies 1, 2 and 3. For this second step, all the data for ferrets 2, 7 and 8 were used, but ferret 1 data from Study 1 were not fitted because the profile fell outside the rest of the population. However, the data for this ferret can be seen in the S1 Table. Data from Studies 1, 2, and 3 were combined into one database including ferrets inoculated with influenza A and B virus as well as uninfected ferrets for the final evaluation during step 2 model fitting. The final database contained 430 OC measurements above LOQ from a total of 65 ferrets: inoculated with influenza A virus (n = 30 total, n = 18 for H5N1 from Study 1 Parts 1 and 2, and n = 12 for H3N2 from Study 1 Part 2), inoculated with influenza B virus (n = 18) from Study 2, and uninfected ferrets (n = 17) from Studies 1, 2 and 3. Of these 65 ferrets, the 57 from Studies 1 and 2 had received ketamine anesthesia prior to blood sampling, while the eight in Study 3 were not administered ketamine prior to blood sampling.

The residual error model included both additive and proportional error. We assumed parameters to be log-normally distributed. Linear PK was assumed.

To understand factors affecting PK, the Kruskal-Wallis test was performed on PK parameters with anesthesia and influenza strains used as covariates. Subsequent pairwise comparison was performed with influenza strain. In all ferrets with fitted PK parameters, the 24-h steady-state AUC (AUC_ss24h_) was calculated by taking the 24-h dose divided by the fitted CL_t_.

### Simulations

Monte Carlo simulations were conducted to better understand PK variability expected for a typical PK/PD study. For these simulations, influenza A and B study data were pooled and iterative two-stage analysis was performed with a full covariance matrix. The resulting parameter means and covariance matrix were used to simulate OC PK in 1000 ferrets, with the following modifications: CV% for V_c_, Cl_d_, and K_t_ were empirically reduced to 50%, and all related covariance terms were fixed to zero; any other covariance terms with correlation <0.15 were also fixed to zero. The empirical reduction in CV% was necessary due to an overestimation of PK variability apparent in a visual predictive check without this change. The dose used was 1.0, 5.0, and 25.0 mg of oral OP q12h for 5 days (typical doses that might be used for a PK/PD study). PK parameters were calculated using concentrations from 12 h after the last dose.

A separate Monte Carlo simulation with 100 ferrets was performed to simulate median PK and the 80% confidence interval at the dose that results in the same median OC AUC_ss12h_ observed in humans during steady state at the approved dose of 75 mg q12h, 3220 ng h/mL [6,19]. The oral dose simulated was 3.87 mg/kg of OFB (5.08 mg/kg of OP) q12h for 5 days. Simulated steady-state concentrations were taken on the 5th day during the last dose.

## Results

The PK data for OP and OC in a ferret animal model presented here highlight a potential influence of anesthesia on OP and OC PK parameters. A novel ferret PK model for OC was developed from these data. Clinical signs of influenza virus infected animals are described to characterize the disease severity. Additionally, the population PK model was used to examine whether OC PK is affected by anesthesia and the influenza virus strain used to challenge animals.

### Population PK Model

Before initiating modeling, an overview of available PK data was conducted to determine which PK data would prove useful for modeling. NCA was conducted for a similar dose level in Studies 1–4 to determine whether there were obvious differences between data sets (Table 1). Studies 1–3 all showed similar PK parameters, including a T_max_ of 1 h for OP and of 3–4 h for OC. Study 4, with animals maintained under Saffan anesthesia, exhibited a clear difference with higher exposures to OC (AUC_0-12hr_ ~1.8- to ~2.8-fold higher in Study 4 ferrets compared with ferrets in other studies; Table 1) and delayed bioavailability, with a T_max_ of 7 h for both OP and OC indicating delayed absorption of OP. The differences in PK between Study 4 and the other studies led to the decision to leave Study 4 data out of the PK model. However, PK differences are sometimes difficult to identify in data analyzed by NCA, particularly when comparing data generated by different study designs as done here. Therefore, given the difference in the group maintained under Saffan anesthesia, the strategy for model building was to include data from studies 1–3, and then to determine whether the PK was similar or different in the studies with ketamine versus no anesthesia as part of the model-building process.

**Table 1:** OP and OC PK parameters in uninfected ferrets following an oral dose of oseltamivir.^a^

Study	Anesthesia	Dose, mg/kg^b^	Number of ferrets / dose	PK parameters^c^					
				C_max_, μg/mL		T_max_, h		AUC_0–12h_, μg h/mL	
				OP	OC	OP	OC	OP	OC
Study 1	Ketamine^d^	5.0	3	1.42 ± 0.81	0.45 ± 0.47	1 ± 0.3	3 ± 2	4.20 ± 0.42	2.61 ± 1.99
Study 2	Ketamine^d^	3.8	6	1.04	0.54	1	4	2.53	2.78
Study 3	None	3.8	4	1.10 ± 0.52	0.58 ± 0.35	1 ± 0.6	3 ± 1	3.87 ± 1.47	4.03 ± 1.63
Study 4	Saffan^e^	5.0	4	0.74 ± 0.34	1.16 ± 0.27	7 ± 6	7 ± 4	2.82 ± 0.80	7.40 ± 3.44

^a^ This PK analysis used data from uninfected animals (e.g., from Study 1, uninfected ferrets with rich PK data).

^b^ The dose was calculated based on free-base oseltamivir molecular weight.

^c^ Except for Study 2, PK parameters are reported as mean ± SD. For Study 2 this was not possible because the data had to be treated as composite data.

^d^ Ketamine was administered at time of blood collection from ferrets.

^e^ Animals were maintained on Saffan anesthesia during the PK experiment.

AUC_0–12h_ = area under the plasma concentration-time curve from 0–12 h; C_max_ = maximum observed plasma concentration; OC = oseltamivir carboxylate; OP = oseltamivir phosphate; PK = pharmacokinetic; T_max_ = time to reach the maximum observed plasma concentration.

**Fig 3:**
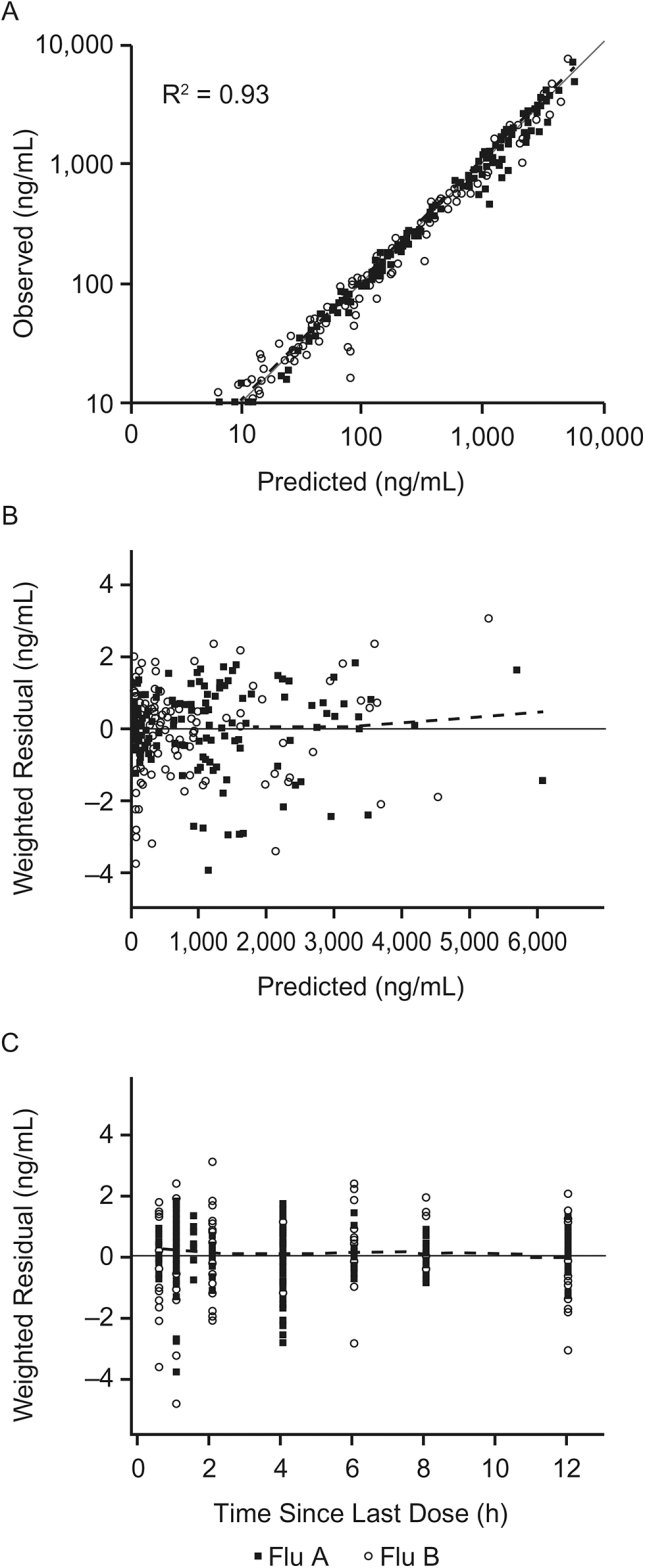
Diagnostic plots for the PK model from influenza-inoculated ferret data from studies 1 and 2. Closed squares and open circles are for animals inoculated with influenza A and influenza B, respectively. Diagnostic plots include (A) predicted versus observed, (B) weighted residuals versus predicted, and (C) weighted residuals versus time since the last dose. The dashed lines are loess curves.

**Fig 4:**
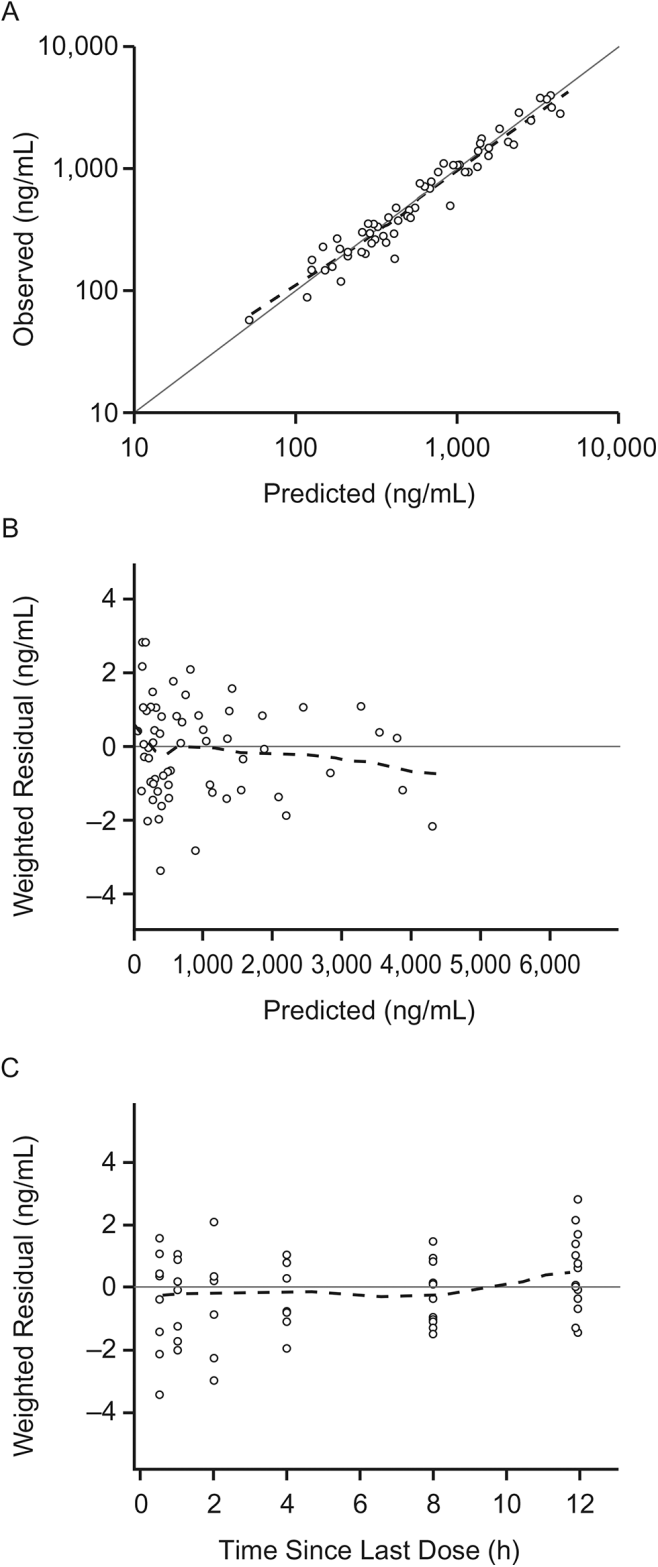
Diagnostic plots for the PK model from uninfected ferret data from studies 1 to 3. Diagnostic plots include (A) predicted versus observed, (B) weighted residuals versus predicted, and (C) weighted residuals versus time since the last dose. The dashed lines are loess curves.

**Fig 5:**
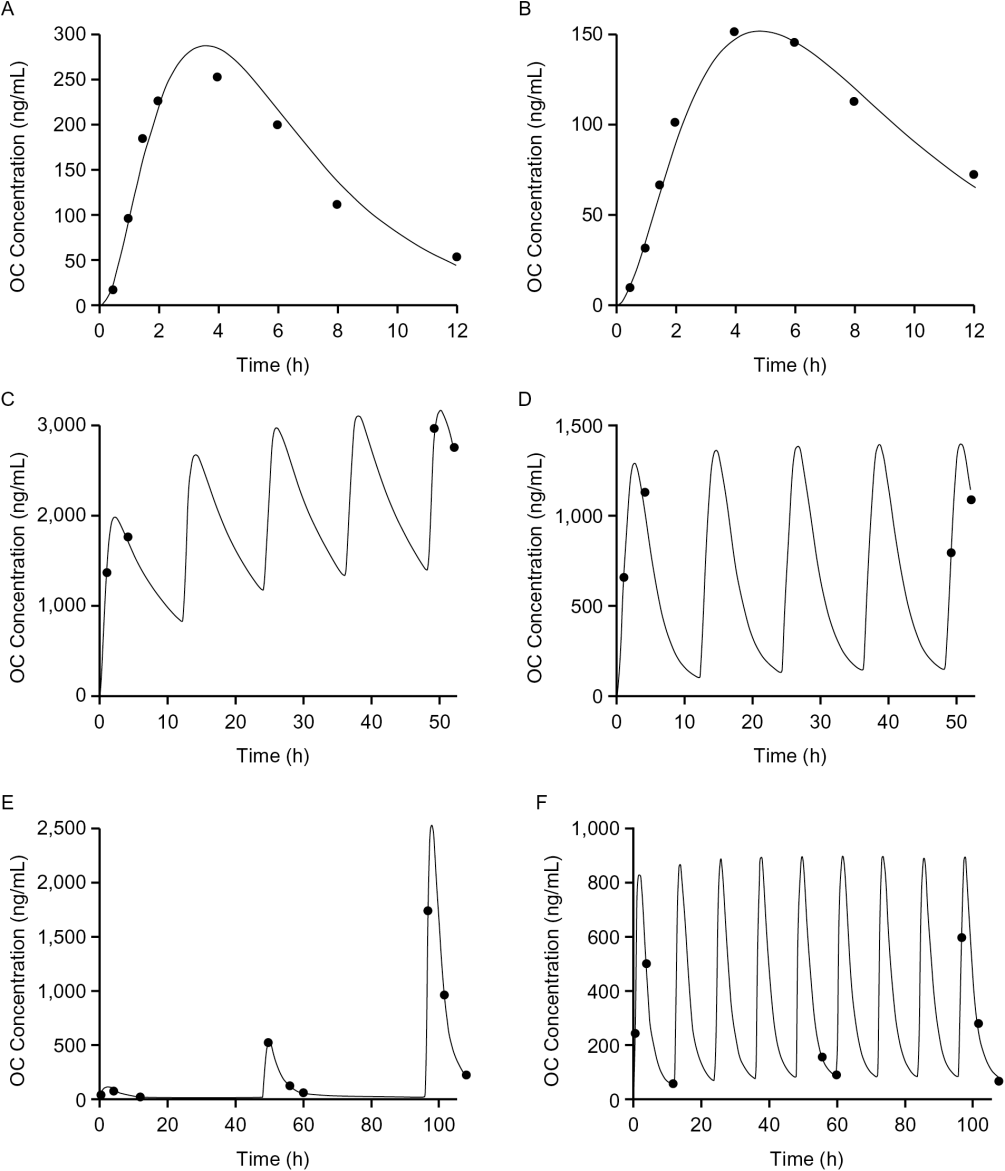
Post-hoc fits in representative ferrets. Observed and simulated OC PK in representative ferrets from studies 1 and 2: (A) rich PK in an H5N1-inoculated ferret administered a 12.5 mg/kg OP dose; (B) rich PK in an uninfected ferret administered a 5.0 mg/kg OP dose; (C) sparse PK in an H5N1-infected ferret administered 25.0 mg/kg OP q12h; (D) sparse PK in an H3N2-infected ferret administered 12.5 mg/kg OP every twelve h (q12h); (E) PK for an uninfected ferret in the PK control group administered 0.76, 3.8, and 19.0 mg/kg OP on days 1, 3, and 5, respectively; and (F) sparse PK in a ferret inoculated with influenza B/Yamagata/16/1988 influenza virus administered 3.8 mg/kg OP q12h.

The ferret OC PK data from studies 1, 2, and 3 were well-described using a population PK model incorporating delayed first-order input, two-compartment distribution, and first-order elimination. The overall R^2^ of the model was 0.93, and the model performed well with no bias (Figs 3 and 4). Residuals were evenly distributed throughout the entire range of concentrations and time, and there was no bias due to the influenza strain used to challenge ferrets. The post-hoc fits showed good prediction of OC concentrations. Figures comparing model fits with observed data for representative individual ferrets are shown in Fig 5, and all model fits for all ferrets are provided in S1 Fig. The intercept for the additive error model was 5 ng/mL, roughly equal to half of the LOQ. The slope was 0.15, roughly equal to the coefficient of variation (CV%) of the assay performance.

### Clinical Signs in Influenza Virus-Infected Ferrets

For Study 1, when ferrets were infected with a low dose of influenza A A/Shenzheng/406H/2006 (H5N1) virus, control ferrets showed relatively mild signs of illness: only three out of six animals infected showed at least 1°C increase in body temperature on days 3 and 5 post-infection (p.i.), the animals lost 6.3% of initial body weight on day 5 p.i., and mean activity score was 0.6 (Table 2). The ferrets that received both 12.5 and 25 mg/kg OP stayed active throughout the observation period (mean activity score, 0.1), and showed less weight loss. Administration of 25 mg/kg OP resulted in a pronounced effect on the development of fever (it was detected only in 1 out of 6 H5N1-infected ferrets on day 3 p.i.,Table 2). No ferrets received an activity score of three at any point in the study, and zero was the most common score.

**Table 2:** Selected PK parameters and clinical signs in ferrets challenged with influenza A viruses (Study 1).

Influenza A virus	Virus challenge dose, TCID_50_	OP dose, mg/kg^a^	OC PK parameter^b^ AUC_ss12h_, ug h/mL	Clinical signs and PD parameters^b^				
				Body temperature change on day p.i.,°C^c^			Body weight change on day p.i., %^d^	Activity score^e^
				Day 1	Day 3	Day 5	Day 5	
A/Shenzheng/406H/2006 (H5N1)	10^2^	0	‒^f^	0.3 ± 0.6 (1/6)	0.8 ± 1.0 (3/6)	0.9 ± 0.9 (3/6)	-6.3 ± 11.2 (2/6)	0.6 ± 0.3
	10^2^	12.5	7.0 ± 2.3	0.7 ± 0.4 (1/6)	1.4 ± 0.6 (4/6)	0.3 ± 1.5 (2/6)	1.9 ± 6.37 (1/6)	0.1 ± 0.2
	10^2^	25.0	13.2 ± 9.6	-0.3 ± 1.1 (0/6)	0.5 ± 0.5 (1/6)	-0.3 ± 0.4 (0/6)	-0.8 ± 2.4 (0/6)	0.1 ± 0.2
A/Hong Kong/433581/2009 (H3N2)	10^6^	0	±	0.5 ±± 1.4 (2/6)	0.1 ± 0.8 (1/6)	-0.4 ± 1.2 (1/6)	-4.7 ± 9.0 (1/6)	0.4 ± 0.3
	10^6^	12.5	6.4 ± 1.2	1.4 ± 0.5 (5/6)	-0.8 ± 1.1 (1/6)	0.0 ± 1.7 (2/6)	-2.0 ± 3.1 (2/6)	0.2 ± 0.1
	10^6^	25.0	18.0 ± 7.0	0.6 ± 1.0 (2/6)	-0.5 ± 1.1 (1/6)	0.2 ± 0.9 (1/6)	-3.7 ± 0.5 (0/6)	0.1 ± 0.2

^a^ OP dose was calculated based on free-base oseltamivir molecular weight.

^b^ Values are mean ± SD. All PD parameters were from morning observations.

^c^ Each ferret’s body temperature was measured by subcutaneous implantable encapsulated microchips (IPTT-300, Bio Medic Data Systems, US), which was recorded for 3 days before virus inoculation. The body temperature change was calculated for each ferret individually as an increase or decrease of its temperature on day 0 before virus inoculation. The values are averages for six animals per group (°C) ± SD. The average temperature decrease is indicated by minus. Positive average values indicate that the temperature increased. The number of ferrets per the total number of animals in that group that had at least a 1°C increase in temperature from baseline is shown in parenthesis.

^d^ The weight change was calculated for each ferret individually as a percentage of its weight on day 0 before virus inoculation. The values are averages for six animals per group (%) ± SD. The average weight loss is indicated by a minus sign. Positive average values indicate that animals were gaining weight. The number of ferrets per total number of animals in that group that had more than a 5% loss of initial weight on day 5 p.i. is shown in parenthesis.

^e^ The reported value is the mean morning activity score ± standard deviation observed on days 1–5 after inoculation on day 0. A score of zero indicated no nasal symptoms and a fully playful ferret. A score of one indicated nasal rattling or sneezing and a ferret that responded to play overtures but that did not initiate play. A score of two indicated nasal discharge and a ferret that was alert but not playful. A score of three indicated that mouth breathing was necessary and the ferret was not playful or alert.

^f^ Blood samples were not collected as OP was not administered.

AUC_ss12h_ = steady-state 12-h area under the plasma concentration-time curve calculated from the population PK model individual fit CL for each animal: AUC_ss12h_ = D×CL; CL = clearance; OC = oseltamivir carboxylate; OP = oseltamivir phosphate; PD = pharmacodynamic; p.i. = post-infection; PK = pharmacokinetic; SD = standard deviation; TCID_50_ = 50% tissue culture infectious dose.

The ferrets infected with A/Hong Kong/433581/2009 (H3N2) influenza virus also developed mild infection with mean activity score of 0.4 (Table 2). Animals treated with both 12.5 and 25 mg/kg OP showed a mean weight loss of 2.0% and 3.7% respectively, and remained active (Table 2).

Inoculation of ferrets with B/Yamagata/16/1988 influenza virus (Study 2) caused mild infection: no pronounced changes were observed in body weight, body temperature, or relative activity (relative inactivity index of 0.2 for all oseltamivir-treated groups) of ferrets across all groups throughout the study (Table 3). In all influenza B virus inoculated groups, animals did not lose weight and on average a weight gain was observed. Mild respiratory clinical signs in four out of six animals were observed in all the oseltamivir treatment and uninfected groups.

**Table 3:** Selected PK parameters and clinical signs in ferret challenged with influenza B virus (Study 2).

Influenza B virus	Virus challenge dose, PFU	OP dose, mg/kg^a^	OC PK parameter^b^ AUC_ss12h_, ug h/mL	PD parameters (clinical signs)^b^				
				Body temperature change on day p.i.,°C^c^			Body weight change on day p.i., %^d^	Activity score^e^
				Day 1	Day 3	Day 5	Day 5	
B/Yamagata/16/1988	0	0.76^f^	0.63 ± 0.44	0.0 ± 0.9 (1/6)	‒^f^	‒^f^	‒^f^	0.3 ± 0.0
	0	3.8^f^	3.33 ± 2.11	‒^f^	0.6 ± 1.0 (2/6)	‒^f^	‒^f^	0.3 ± 0.0
	0	19^g^	16.39 ± 11.41	‒^f^	‒^f^	-0.1 ± 0.6 (1/6)	0.7 ± 0.7 (2/6)	0.3 ± 0.0
	10^7^	0	‒^h^	1.1 ± 0.7 (2/5)	0.8 ± 0.6 (2/5)	0.9 ± 0.7 (3/5)	3.3 ± 5.2 (0/5)	0.2 ± 0.0
	10^7^	0.76	0.38 ± 0.06	0.4 ± 1.1 (3/6)	0.9 ± 0.5 (3/6)	0.9 ± 1.1 (2/6)	5.1 ± 8.0 (0/6)	0.2 ± 0.0
	10^7^	3.8	2.46 ± 0.39	1.2 ± 1.0 (4/6)	1.3 ± 0.4 (4/6)	0.9 ± 0.9 (2/6)	8.3 ± 4.4 (0/6)	0.2 ± 0.0
	10^7^	19	14.10 ± 2.85	0.5 ± 0.3 (1/6)	0.5 ± 0.7 (2/6)	0.3 ± 0.6 (1/6)	6.1 ± 3.9 (0/6)	0.2 ± 0.0

^a^ OP dose was calculated based on free-base oseltamivir molecular weight.

^b^ Values are mean ± SD. All PD parameters were from morning observations.

^c^ Each ferret’s body temperature was measured by subcutaneous implantable temperature transponders (Bio Medic Data Systems Inc., Seaford, DE, United States of America), and was recorded for 3 days before virus inoculation. The values were averaged to obtain a baseline value. The body temperature change was calculated for each ferret individually as an increase or decrease of its temperature on day 0 before virus inoculation. The values are averages for 5–6 animals per group (°C) ± SD. The average temperature decrease is indicated by minus. Positive average values indicate that temperature increased. The number of ferrets per the total number of animals in that group that had at least a 1°C increase in temperature from baseline is shown in parenthesis.

^d^ The weight change was calculated for each ferret individually as a percentage of its weight on day 0 before virus inoculation. The values are averages for 5–6 animals per group (%) ± SD. Positive average values indicate that animals were gaining weight. The number of ferrets per total number of animals in that group that had more than a 5% loss of initial body weight on day 5 p.i. is shown in parenthesis.

^e^ The reported value is the mean morning activity score ± SD observed on days 1–5 after inoculation on day 0. A score of zero indicated no nasal symptoms and a fully playful ferret. A score of one indicated nasal rattling or sneezing and a ferret that responded to play overtures but that did not initiate play. A score of two indicated nasal discharge and a ferret that was alert but not playful. A score of three indicated that mouth breathing was necessary and the ferret was not playful or alert.

^f^ These data are from the group of ferrets administered a different dose on day 1, 3, and 5 to obtain PK data.

^g^ This group is the uninfected PK control that received a 0.76, 3.8, and 19.0 mg/kg OP dose on days 1, 3, and 5, respectively.

^h^ Blood samples were not collected as OP was not administered.

AUC_ss12h_ = steady-state 12-h area under the plasma concentration-time curve calculated from the population PK model individual fit CL for each animal: AUC_ss12h_ = D×CL; CL = clearance; OC = oseltamivir carboxylate; OP = oseltamivir phosphate; PD = pharmacodynamic; PFU = plaque forming units; p.i. = post-infection; PK = pharmacokinetic, SD = standard deviation.

### Factors Affecting OC PK

Maintaining ferrets under anesthesia had a clear effect on PK that was apparent from NCA (Table 1), and even ketamine anesthesia seemed to affect OC PK. The Kruskal-Wallis test applied to parameter estimates (Table 4) showed that there was a statistically significant difference in all the model parameters (except for CL_d_): K_t_, and V_c_ decreased and K_a_, CL_t_, and V_p_ increased in the pooled analysis from Studies 1 and 2 (ketamine anesthesia was used before plasma samples were drawn) when compared with the pooled analysis from Study 3 (no anesthesia).

**Table 4:** OC PK parameters estimated in ferrets with and without anesthesia.

Parameter	Studies 1 and 2, ketamine		Study 3, no anesthesia		Kruskal-Wallis Test (*p*-value)
	Mean	CV%	Mean	CV%	
K_t_ (h^-1^)	1.27	79.8	4.18	42.5	<0.001
K_a_ (h^-1^)	0.463	66.1	0.335	37.8	0.01
CL_d_ (L/h)^a^	0.585	96.6	0.878	23.7	0.174
CL_t_ (L/h)^a^	1.52	55.1	0.919	16.5	0.001
V_c_ (L)^a^	0.157	165	1.00	66	0.002
V_p_ (L)^a^	5.59	63.6	2.08	33.6	<0.001

^a^ The CL and V parameters are conditioned on oral bioavailability and fraction of parent changed to metabolite.

CL_d_ = distribution clearance; CL_t_ = first-order clearance from the central compartment; CV = coefficient of variation; K_a_ = first-order absorption rate constant; K_t_ = first-order transfer rate constant for transit compartments; OC = oseltamivir carboxylate; PK = pharmacokinetic; V_c_ = central compartment volume; V_p_ = peripheral compartment volume.

Influenza virus used to challenge ferrets did not affect PK parameters (Table 5). Even though there was a statistically significant difference in K_a_ and CL_d_ due to inoculation strain, subsequent pairwise comparisons failed to show any difference amongst the four groups. Therefore, at least within these studies with subclinical influenza, inoculation strain did not affect PK.

**Table 5:** OC PK parameters estimated in uninfected and influenza virus-infected ferrets.

Parameter	Uninfected		Influenza A and B viruses used to challenge ferrets:						Kruskal-Wallis test (*p*-value)
			A/Shenzheng/406H/2006 (H5N1)		A/Hong Kong/433581/2009 (H3N2)		B/Yamagata/16/1988		
	Mean	CV%	Mean	CV%	Mean	CV%	Mean	CV%	
K_t_ (h^-1^)	1.18	34.5	1.20	46.7	1.24	28.9	1.56	24.0	0.086
K_a_ (h^-1^)	0.526	90.3	0.287	72.6	0.552	22.9	0.498	17.9	0.012
CL_d_ (L/h)^a^	0.774	48.9	1.091	56.8	0.501	54.6	0.386	48.1	<0.01
CL_t_ (L/h)^a^	1.54	50.8	1.505	78.0	1.46	26.3	1.35	21.9	0.597
V_c_ (L)^a^	0.386	40.7	0.312	13.4	0.312	54.1	0.335	38.4	0.713
V_p_ (L)^a^	6.45	43.8	9.24	21.5	8.11	30.9	8.68	52.9	0.086

^a^ The CL and V parameters are conditioned on oral bioavailability and fraction of parent changed to metabolite.

CL_d_ = distribution clearance; CL_t_ = first-order clearance from the central compartment; CV = coefficient of variation; K_a_ = first-order absorption rate constant; K_t_ = first-order transfer rate constant for transit compartments; OC = oseltamivir carboxylate; PK = pharmacokinetic; Vc = central compartment volume; Vp = peripheral compartment volume.

### Variability of OC PK

**Fig 6:**
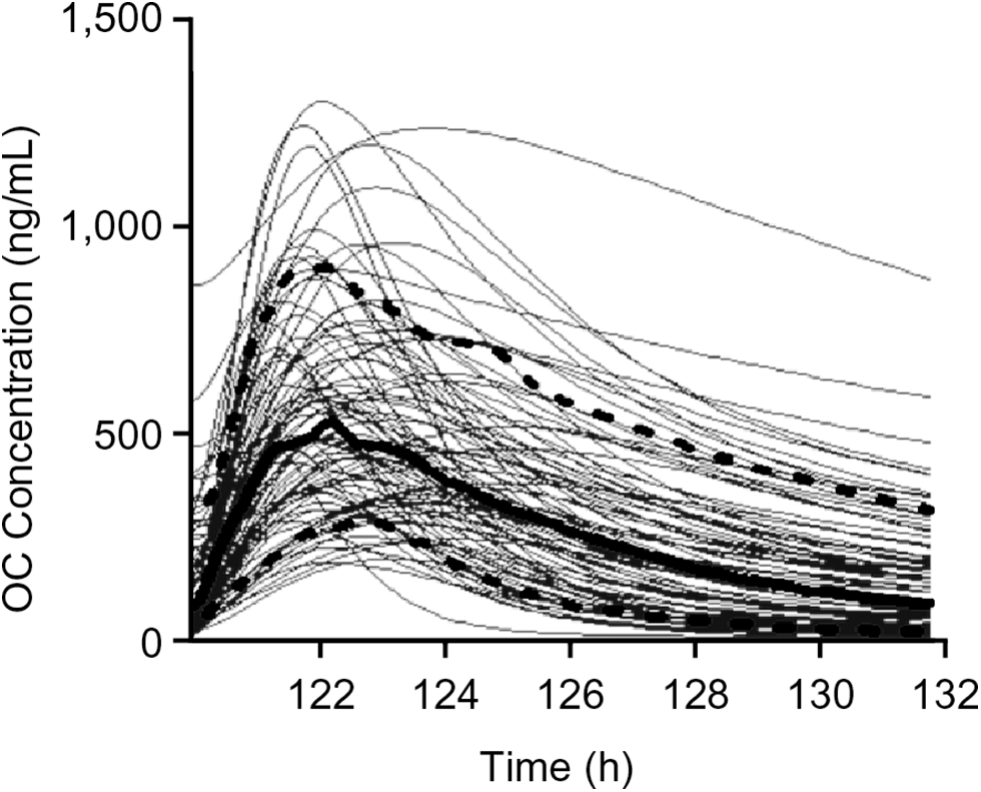
Monte Carlo simulation of steady-state OC PK at a 5.08 mg/kg OP dose. The thin solid curves are simulated PK for 100 individual ferrets, the open curves are the 10th–90th percentile, and the thick solid curve is the median OC concentration-time profile.

The parameter mean and covariance matrix is shown in (Table 6). The Monte Carlo simulation of an oral dose at 5.08 mg/kg OP q12h showed wide variability in concentration-time profiles (Fig 6). This was the ferret dose that achieved the same human equivalent AUC_ss12h_ at the approved 75 mg q12h. The Monte Carlo simulation indicates that there was high variability in minimum concentration (C_min_), AUC_ss24h_, C_max,_ and T_max_ (CV% of 100, 57, 45, and 34, respectively, Table 7).

**Table 6:** Monte Carlo simulation parameters and covariance matrix for the OC PK model.

Parameter	Diagonals		Off-diagonals	
	Mean	Variance	Parameters	Covariance
K_t_ (h^-1^)	1.27	0.402	CL_t_, K_a_	0.104
K_a_ (h^-1^)	0.463	0.0937	V_p_, K_a_	-0.636
CL_d_ (L/h)^a^	0.585	0.0856	V_p_, CL_t_	-0.863
CL_t_ (L/h)^a^	1.52	0.704		
V_c_ (L)^a^	0.157	0.00615		
V_p_ (L)^a^	5.59	12.6		

^a^ The CL and V parameters are conditioned on oral bioavailability and fraction of parent changed to metabolite.

CL_d_ = distribution clearance; CL_t_ = first-order clearance from the central compartment; K_a_ = first-order absorption rate constant; K_t_ = first-order transfer rate constant for transit compartments; OC = oseltamivir carboxylate; V_c_ = central compartment volume; V_p_ = peripheral compartment volume.

**Table 7:** OC PK parameters estimated from Monte Carlo simulations for ferrets inoculated with influenza A or B viruses.

OP dose (mg/kg)^b^	OC PK parameters							
	T_max_ (h)		C_max_ (μg/L)		C_min_ (μg/L)		AUC_ss24h_ (μg h/L)^a^	
	Mean (median)	CV (%)	Mean (median)	CV (%)	Mean (median)	CV (%)	Mean (median)	CV (%)
1.0	2.76 (2.6)	33.7	0.109 (0.098)	44.9	0.029 (0.018)	100	1200 (1060)	56.5
5.0	2.76 (2.6)	33.7	0.544 (0.494)	44.9	0.134 (0.090)	100	5990 (5310)	56.5
25.0	2.76 (2.6)	33.7	2.72 (2.47)	44.9	0.671 (0.460)	100	30000 (26600)	56.5

^a^ Calculated by taking the 24-h dose divided by the fitted CL_t_.

^b^ OP dose of 1.0 mg/kg corresponds to 0.76 mg/kg, 5.0 mg/kg to 3.8 mg/kg, and 25.0 mg/kg to 19.0 mg/kg of free-base oseltamivir.

AUC_ss24h_ = steady-state 24-h area under the concentration-time curve; C_max_ = maximum plasma concentration; C_min_ = minimum plasma concentration; CL_t_ = first-order clearance from the central compartment; CV = coefficient of variation; OC = oseltamivir carboxylate; OP = oseltamivir phosphate; PK = pharmacokinetic; T_max_ = time to reach the maximum plasma concentration.

## Discussion

This paper presents ferret data from a range of studies performed at different times in different laboratories to understand oseltamivir PK in ferrets and determine what could be learned about PK/PD study design. For this reason there are differences in study design and drug administration. Meta-analysis techniques were used to combine these data to learn about OC PK.

This study presents several important and useful results. A population PK model for OC is presented that can be used to simulate regimens to aid in design of a PK/PD study. The influence of anesthesia on OC PK was identified. The challenges of PK/PD studies for influenza infections with mild symptoms were illustrated. Also, it was shown that influenza infection with mild symptoms does not affect PK, although a potential effect of influenza infection with more severe symptoms on PK cannot be ruled out.

### Population PK Model Structure

Since the prodrug OP was neither active nor toxic, it could be excluded from the modeling; it was the pharmacologically active OC concentration that was important for PK/PD assessment. Initial exploratory modeling indicated that including OP explicitly in the model as well as OC, an approach that has been adopted by others [21,22,23], results in a PK model that is technically challenging and time-consuming to develop; variability was overestimated, resulting in unrealistic PK simulations. The simplified model structure proposed here provided a good fit to the OC PK data, with transit compartments acting to model both the delay of the OC appearance and the conversion from prodrug to active metabolite. Though there was no definitive reason for choosing to have two transit compartments, this number adequately captured the PK characteristics. This simplified PK model structure saves computational time with no penalty in terms of quality of agreement with OC PK data.

There was high variability in the OP PK parameters for the ferret model even though prodrug was not included in the model. The simulations showed high variability in the PK profiles (Fig 6, Table 7). However, the variability was still lower than it would have been had the OP been explicitly included in the model. Despite the variability, this PK model adequately described OC kinetics. The OC concentration was accurately predicted in most ferrets, with minimal bias (Figs 3 and 4). This simple and robust PK model is useful for simulations, optimal sampling calculations, and further PK/PD modeling. This simplified PK model structure may also prove useful for analysis of clinical studies.

### Implications of Mild Influenza Symptoms

This work was done to improve methods of PK/PD modeling of oseltamivir in the ferret. In previous studies of oseltamivir efficacy in the ferrets infected with highly pathogenic A/Vietnam/1203/2004 (H5N1) influenza virus, many ferrets in the control group and some in the oseltamivir-treated groups died [29], limiting the ability to do PK/PD analysis. The PK/PD studies (Studies 1 and 2) were designed so that animals infected with H5N1 virus would not die, and thereby would provide more time-course data for the analysis. Due to this limitation of the current study, additional experiments could address how altered liver and kidney function resulting from H5N1 virus infection influence plasma levels of OC. The three different influenza viruses used for inoculation of ferrets (Studies 1 and 2) produced minimal clinical symptoms in terms of changes in temperature, body weight, and activity compared with uninfected control animals. For the influenza B/Yamagata/16/1988 and the seasonal influenza A/Hong Kong/433581/2009 (H3N2) strains, high inoculation doses were used (i.e., 10^7^ PFU and 10^6^ TCID_50_ per ferret, respectively), but the strains only caused mild symptoms with PD data that were not significantly different from those of uninfected animals. For the highly pathogenic A/Shenzheng/406H/2006 (H5N1) strain, a lower inoculation dose was used (10^2^ TCID_50_ per ferret) to determine whether it would result in more robust PK/PD data, since with more mild symptoms fewer animals would die and there would theoretically be more time-course data to analyze. Thus, a limitation of the present study is that the influenza symptoms were quite mild even for the H5N1 strain, which minimizes the ability to identify a drug effect.

In Studies 1 and 2, administration of oseltamivir began 24 h after inoculation. The time that drug administration begins after inoculation can influence efficacy [29,37]. However, for the current studies there was little difference in symptoms between inoculated ferrets and healthy ferrets, and therefore even if dosing had begun immediately upon inoculation, the data may have looked similar.

For future oseltamivir PK/PD studies in the ferret, it should be seen as critical to achieve influenza symptoms that are severe enough to identify a drug effect and if pronounced clinical symptoms cannot be achieved for a given influenza strain, it would be prudent to identify an alternative influenza strain. In general, a time-course of symptoms will also be preferred to a simple categorical outcome [38], as time-series data enable more powerful statistical procedures including time-to-event analyses to be applied. The utility of longitudinal symptom data to in viral kinetics modeling has been demonstrated [39].

Although the minimal change from baseline in terms of clinical symptoms limited the utility of these studies for PK/PD assessment, the data were useful for building a population PK model that can be used for PK/PD study design for future studies. But the minor clinical symptoms observed for both Studies 1 and 2 limited the applicability of our results. Our conclusions regarding the PK characteristics of OC apply only for subclinical influenza. For animals with severe influenza symptoms, the conclusion that PK is not influenced by inoculation strain might not hold true.

### Influence of Anesthesia on OC PK

The effect of anesthesia with ketamine on the PK parameters was striking (Table 4). There was a significant decrease in K_t_ due to anesthesia, possibly attributable to effects of the anesthesia on slowing transit times in the ferret. Anesthesia may also increase the extent of distribution and clearance, leading to an increase in both V_p_ and CL_t_. This result means that, for future ferret studies, anesthesia use will have to stay consistent so that the PK of oseltamivir remains unaffected. One caveat of this conclusion regarding anesthesia is that other factors could be driving these differences. The studies were conducted at separate sites with different investigators, study designs (e.g., times of PK sampling were not consistent), solutions for administering oseltamivir (e.g., administration in cat food versus sugar syrup), and ferrets (e.g., from different animal colonies). However, in our opinion the most significant difference between the studies is anesthetic use.

### Importance of PK Sampling in PK/PD Studies

OC PK in ferrets after OP administration is variable, and can be influenced by anesthesia. For example, Monte Carlo simulations indicate that C_min_ values may have a 100% coefficient of variation (Table 7). In terms of PK/PD study design, sparse PK sampling combined with population PK can be used to determine the PK profile of OC in each individual animal in the study, allowing for robust analysis of the PK/PD data. It is acceptable and often required to use anesthesia such as ketamine during a PK/PD study, but its potential impact on OC PK should be considered and potentially mitigated by incorporating sparse PK in the study design.

### Oseltamivir Dose Equivalent to Recommended Human Dose

The median OC AUC_ss12h_ observed in humans during steady state at the approved dose of 75 mg q12h is 3220 ng h/mL [6,19]. Monte Carlo simulations indicate that 5.08 mg/kg OP q12h should achieve the same equivalent human AUC_ss12h_ at the recommended dose of 75 mg twice daily. In a study with ferrets lethally challenged with A/Vietnam/1203/2004 (H5N1) virus, three out of three ferrets inoculated with 10 or 100 EID_50_ with no oseltamivir treatment died, while in ferrets with administration of 2.5 mg/kg OP q12h upon inoculation, only one out of three ferrets inoculated with 10 EID_50_ and none out of three ferrets inoculated with 100 EID_50_ died [29]. In the same study with ferrets lethally challenged with 100 EID_50_ A/Vietnam/1203/2004 (H5N1) virus, three out of three ferrets administered 5.0 mg/kg OP q12h initiated 24 h after inoculation died, but zero out of three ferrets administered 12.5 mg/kg OP q12h initiated 24 h after inoculation died. Therefore, the estimate of 5.08 mg/kg OP q12h seems like a reasonable estimate of the efficacious dose.

## Conclusions

A compartment model including the PK of OC but not OP and consisting of an absorptive compartment, two transit compartments, and a central and peripheral compartment adequately described the PK of OC. It was not necessary to explicitly include the PK of OP in the model to adequately describe the PK of OC, the active species and therefore the species of greater interest. Simulations with this model demonstrated high PK variability. Ketamine anesthesia significantly impacted OC PK after OP administration. Inoculation with influenza did not impact OC PK in ferrets administered OP, but the ferrets included in this study developed subclinical influenza and an influence of disease state on OC PK in cases of severe influenza cannot be ruled out.

## Supporting Information

S1 Fig Model fits to profiles for all individual animals. (PDF)

S1 Table Oseltamivir carboxylate (OC) PK data in csv format. (XLSX)
