# Supplementary material for: Oseltamivir Population Pharmacokinetics in the Ferret: Model Application for Pharmacokinetic/Pharmacodynamic Study Design
Source: PLoS One. 2015 Oct 13;10(10):e0138069. doi: 10.1371/journal.pone.0138069 (PMC4603953; doi:10.1371/journal.pone.0138069)
Supplement: S1 Fig — (PDF) [file pone.0138069.s001.pdf]

S1 Fig. Individual ferret pharmacokinetic data used for model development.

For additional details (e.g., doses that ferrets received), see S1 Table.

Study 1: Ferrets with IDs beginning with TA were infected with H5N1, TB with H3N2 and TC were infected with H5N1, except for ferrets TC-004, TC-005 and TC-008, which were uninfected.

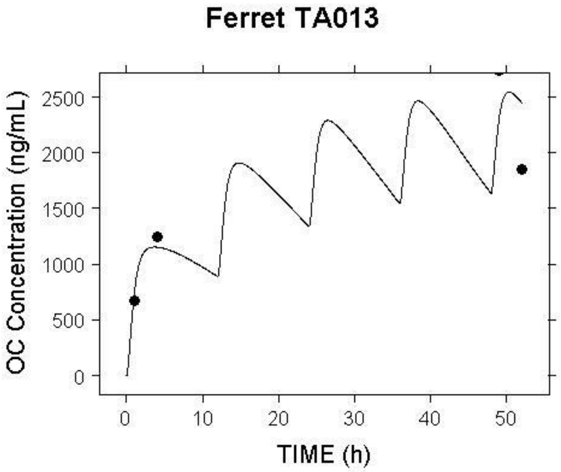

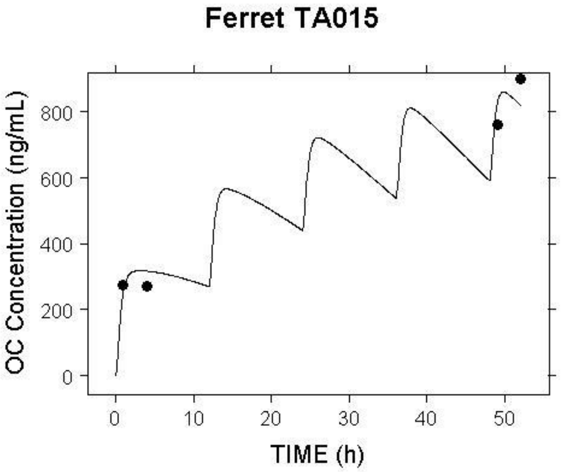

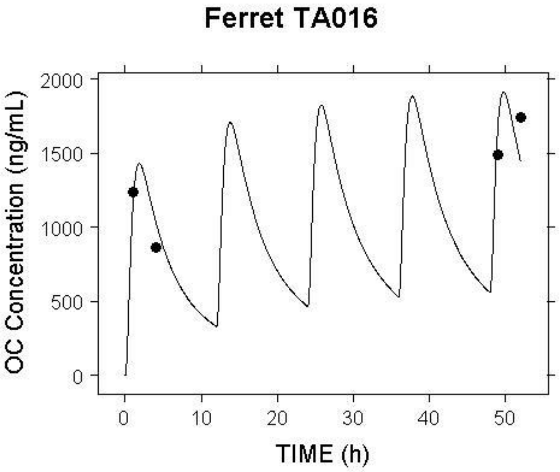

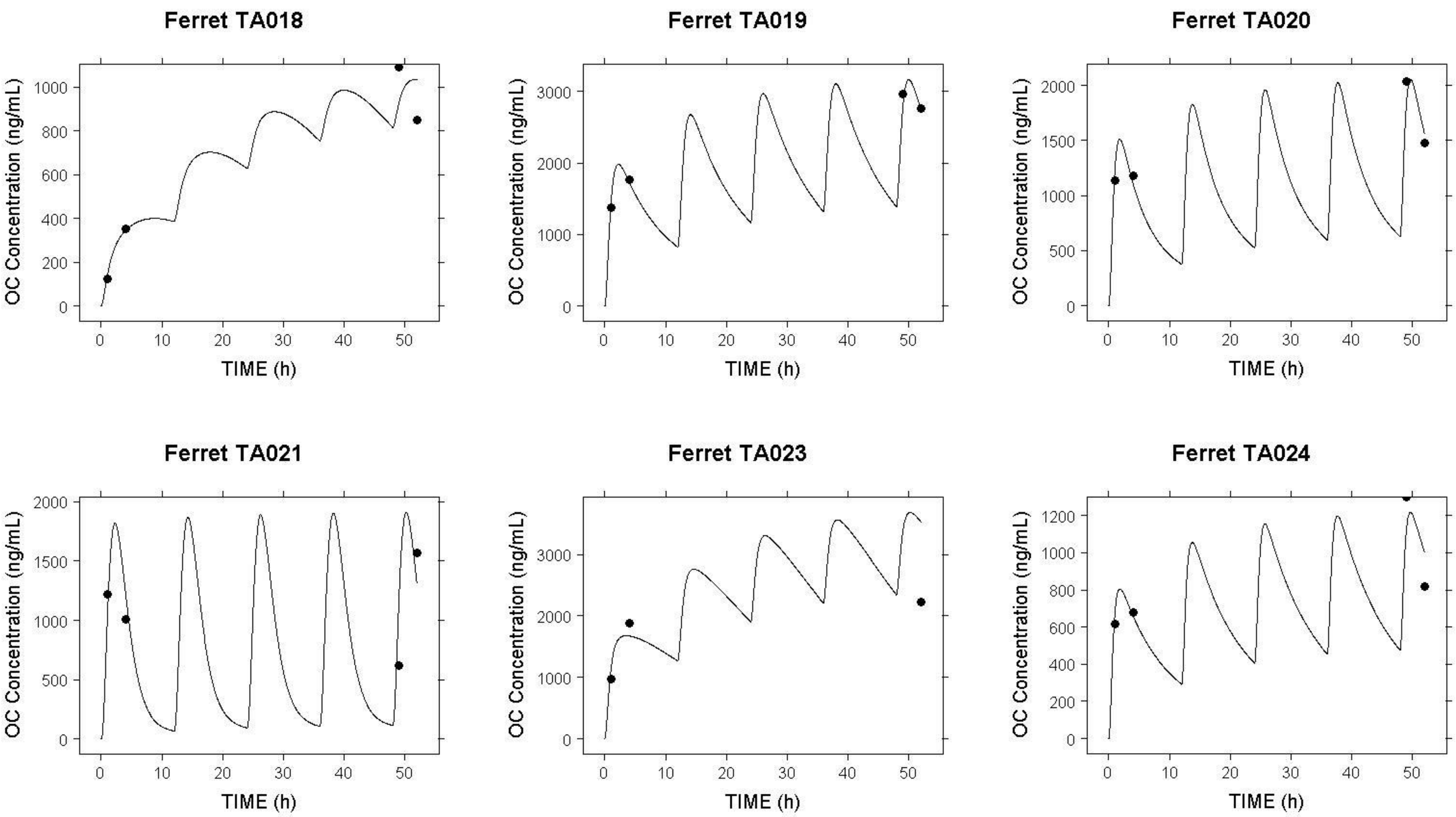
Ferret TA018
Ferret TA019
Ferret TA020
Ferret TA021
Ferret TA023
Ferret TA024
OC Concentration (ng/mL)
TIME (h)
0
10
20
30
40
50
0
200
400
600
800
1000
1000
2000
3000
500
1500
2000
1200

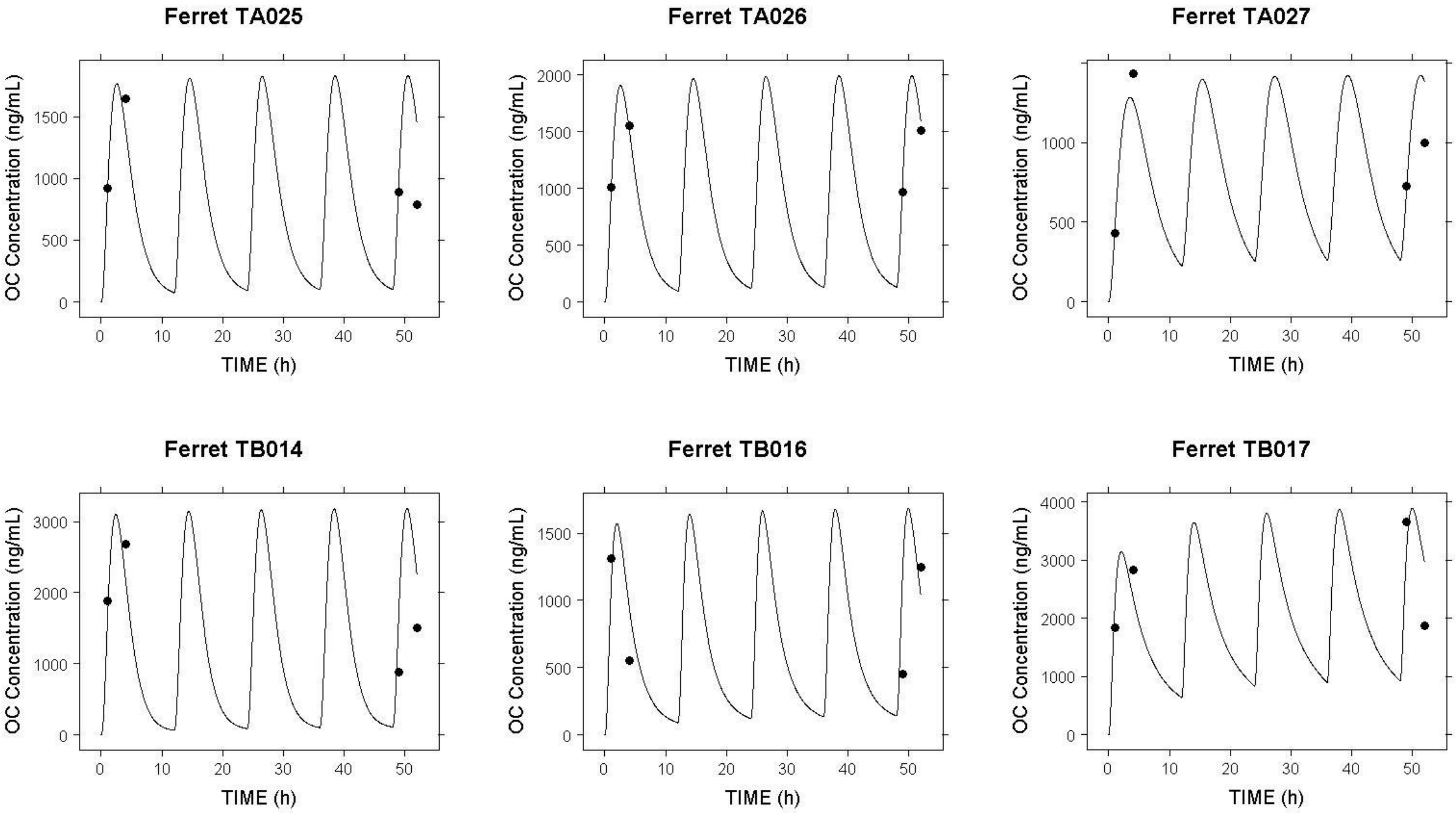
Ferret TA025
Ferret TA026
Ferret TA027
Ferret TB014
Ferret TB016
Ferret TB017
OC Concentration (ng/mL)
TIME (h)
0
10
20
30
40
50
0
500
1000
1500
2000
3000
4000

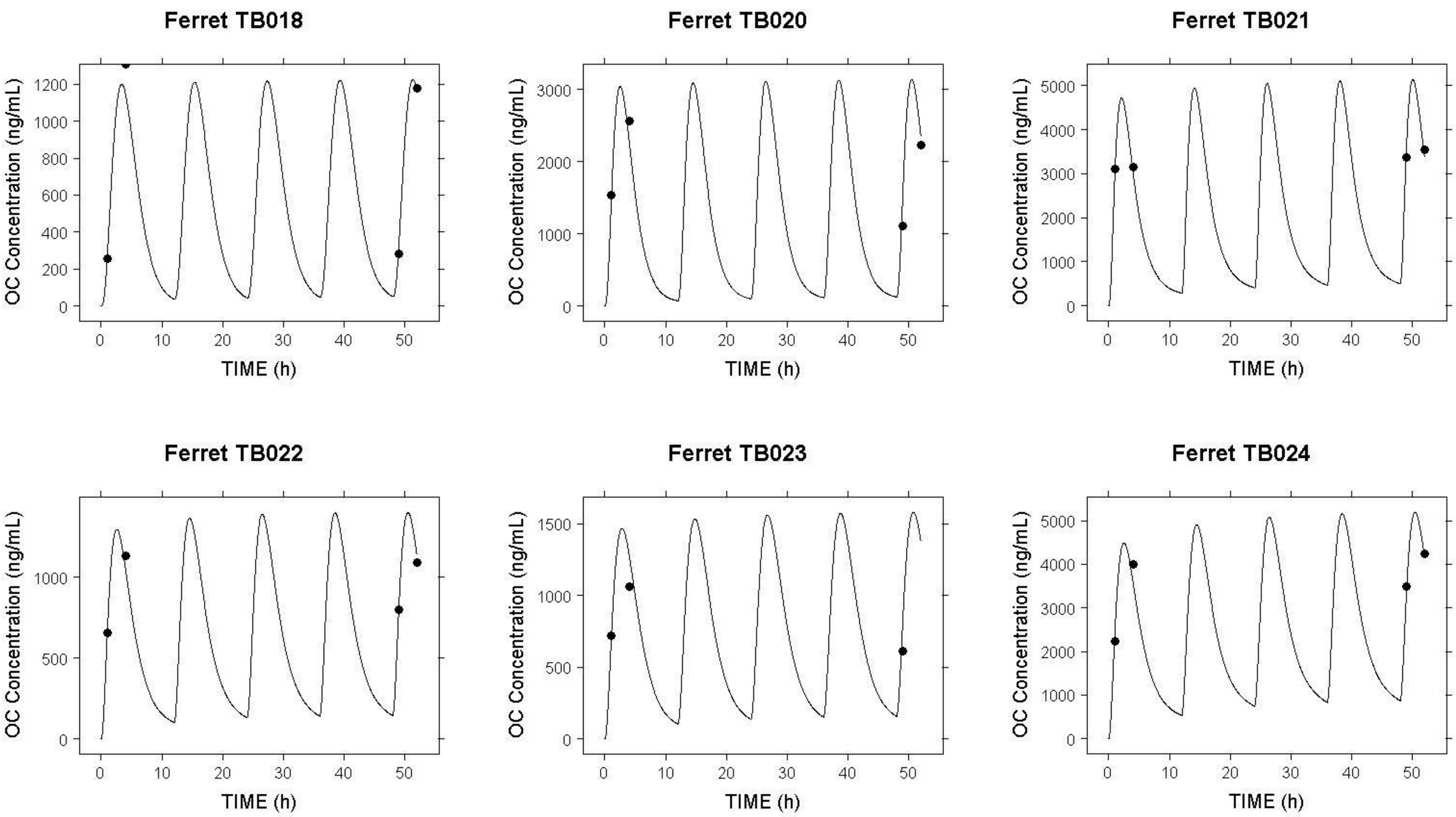
Ferret TB018
OC Concentration (ng/mL)
0
200
400
600
800
1000
1200
TIME (h)
0
10
20
30
40
50
Ferret TB020
OC Concentration (ng/mL)
0
1000
2000
3000
TIME (h)
Ferret TB021
OC Concentration (ng/mL)
0
1000
2000
3000
4000
5000
TIME (h)
Ferret TB022
OC Concentration (ng/mL)
0
500
1000
TIME (h)
Ferret TB023
OC Concentration (ng/mL)
0
500
1000
1500
TIME (h)
Ferret TB024
OC Concentration (ng/mL)
0
1000
2000
3000
4000
5000
TIME (h)

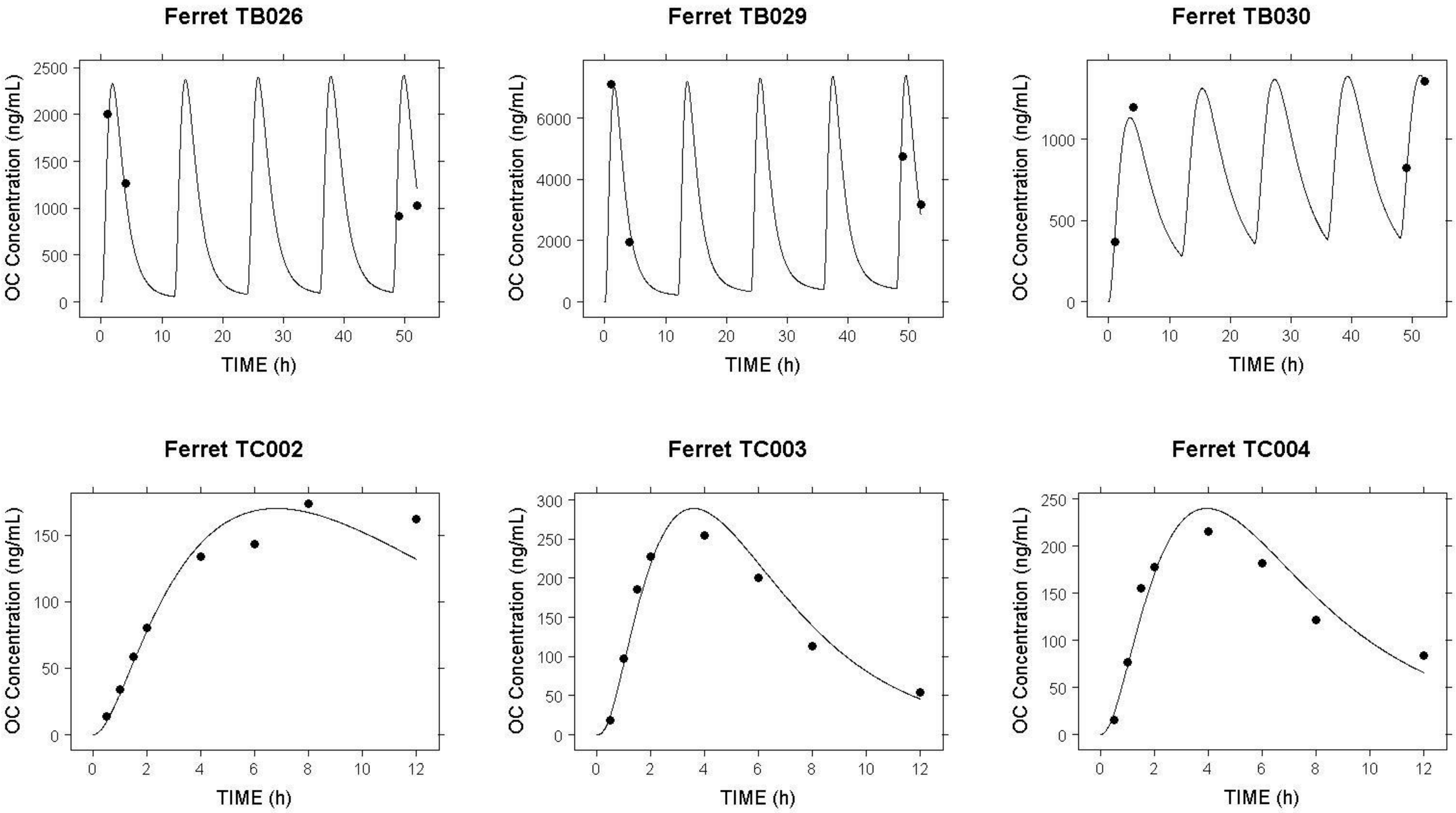
Ferret TB026
Ferret TB029
Ferret TB030
Ferret TC002
Ferret TC003
Ferret TC004
OC Concentration (ng/mL)
TIME (h)

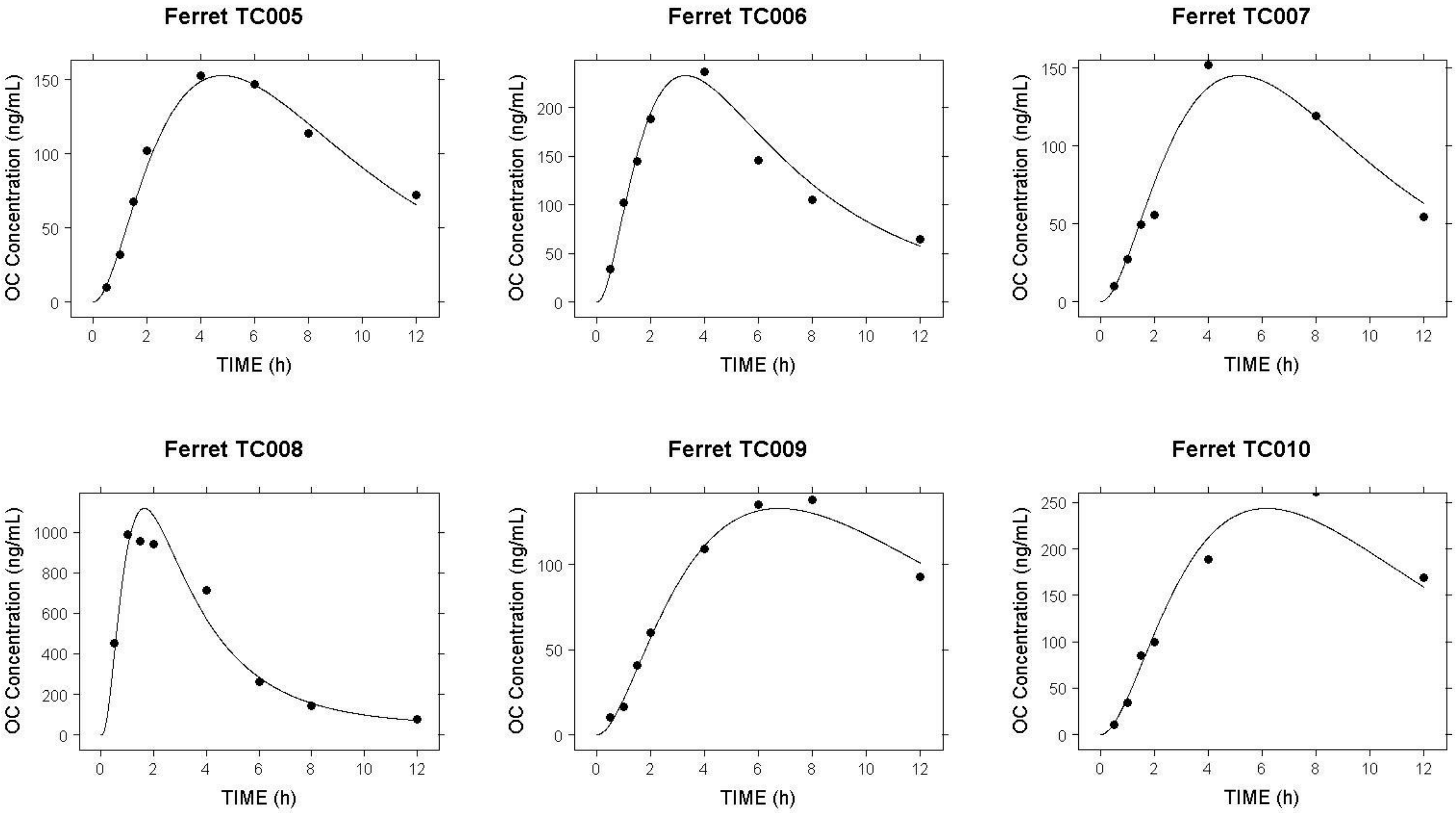

Ferret TC005
Ferret TC006
Ferret TC007
Ferret TC008
Ferret TC009
Ferret TC010
OC Concentration (ng/mL)
TIME (h)
0
2
4
6
8
10
12
0
50
100
150
200
250
400
600
800
1000

Study 2: Ferrets 1-6 were uninfected and ferrets 7-24 were infected with influenza B virus.

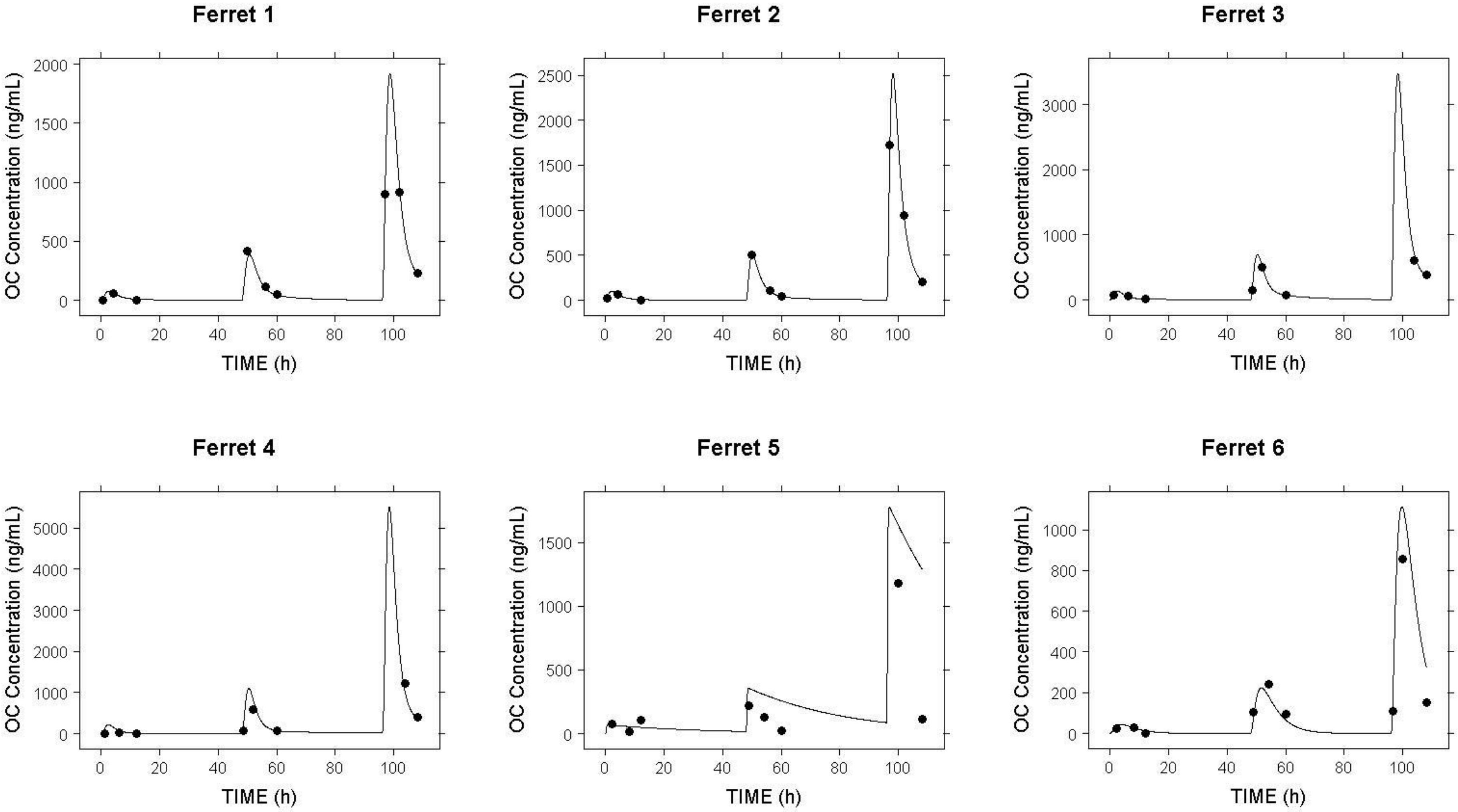

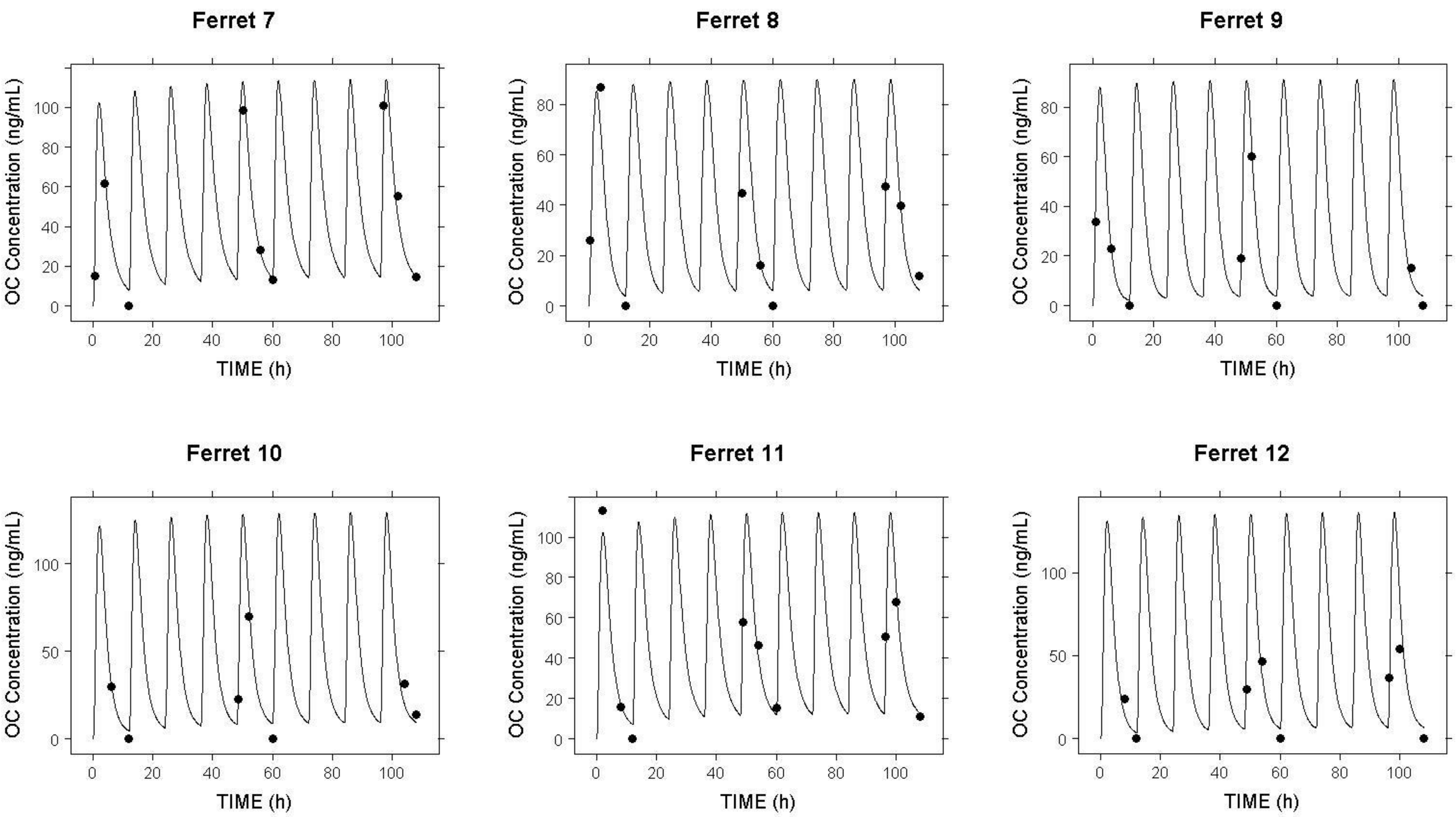
Ferret 7
Ferret 8
Ferret 9
Ferret 10
Ferret 11
Ferret 12
OC Concentration (ng/mL)
TIME (h)
0
20
40
60
80
100
50

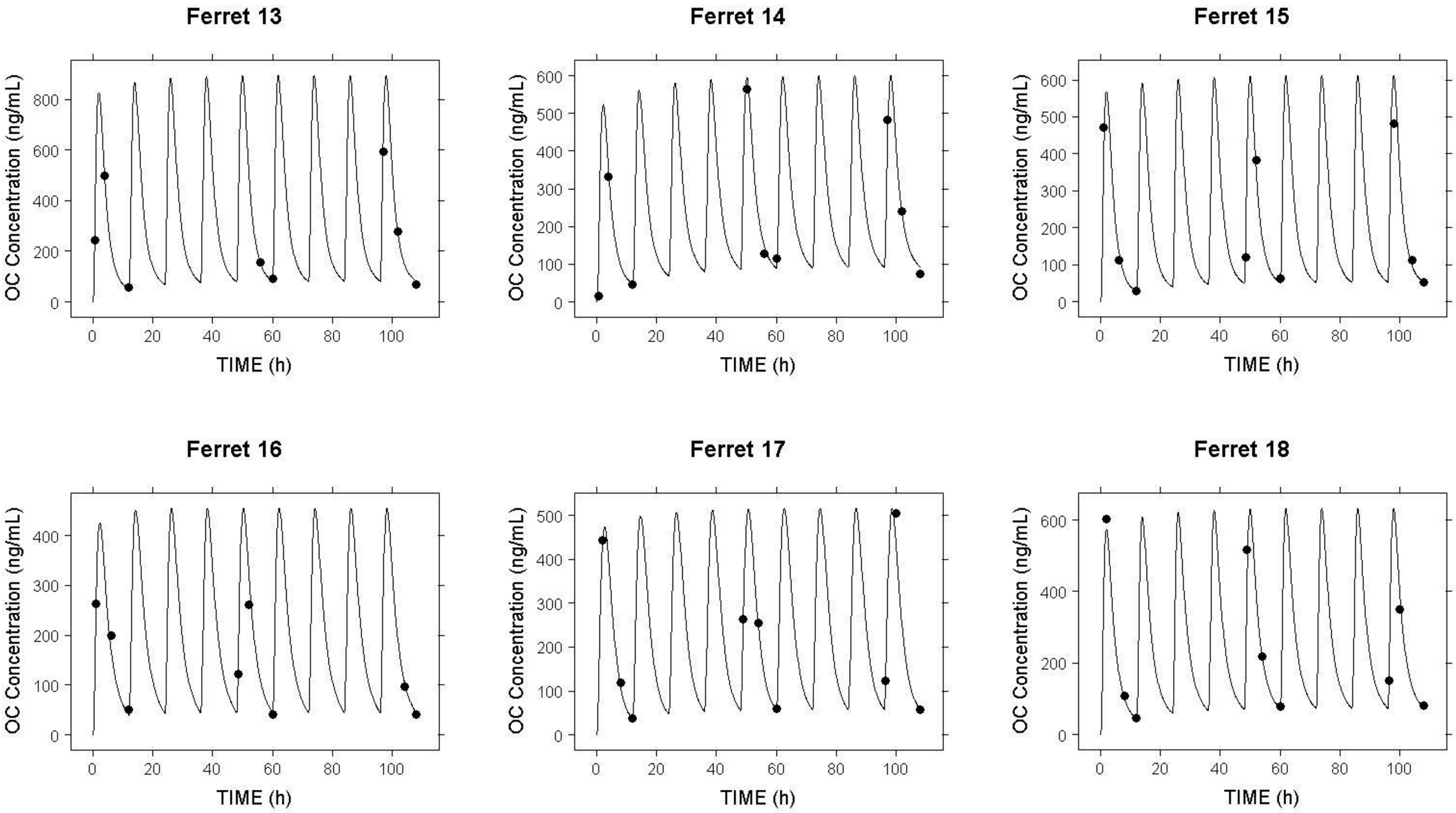
Ferret 13
Ferret 14
Ferret 15
Ferret 16
Ferret 17
Ferret 18
OC Concentration (ng/mL)
TIME (h)
0
20
40
60
80
100
200
300
400
500
600
800

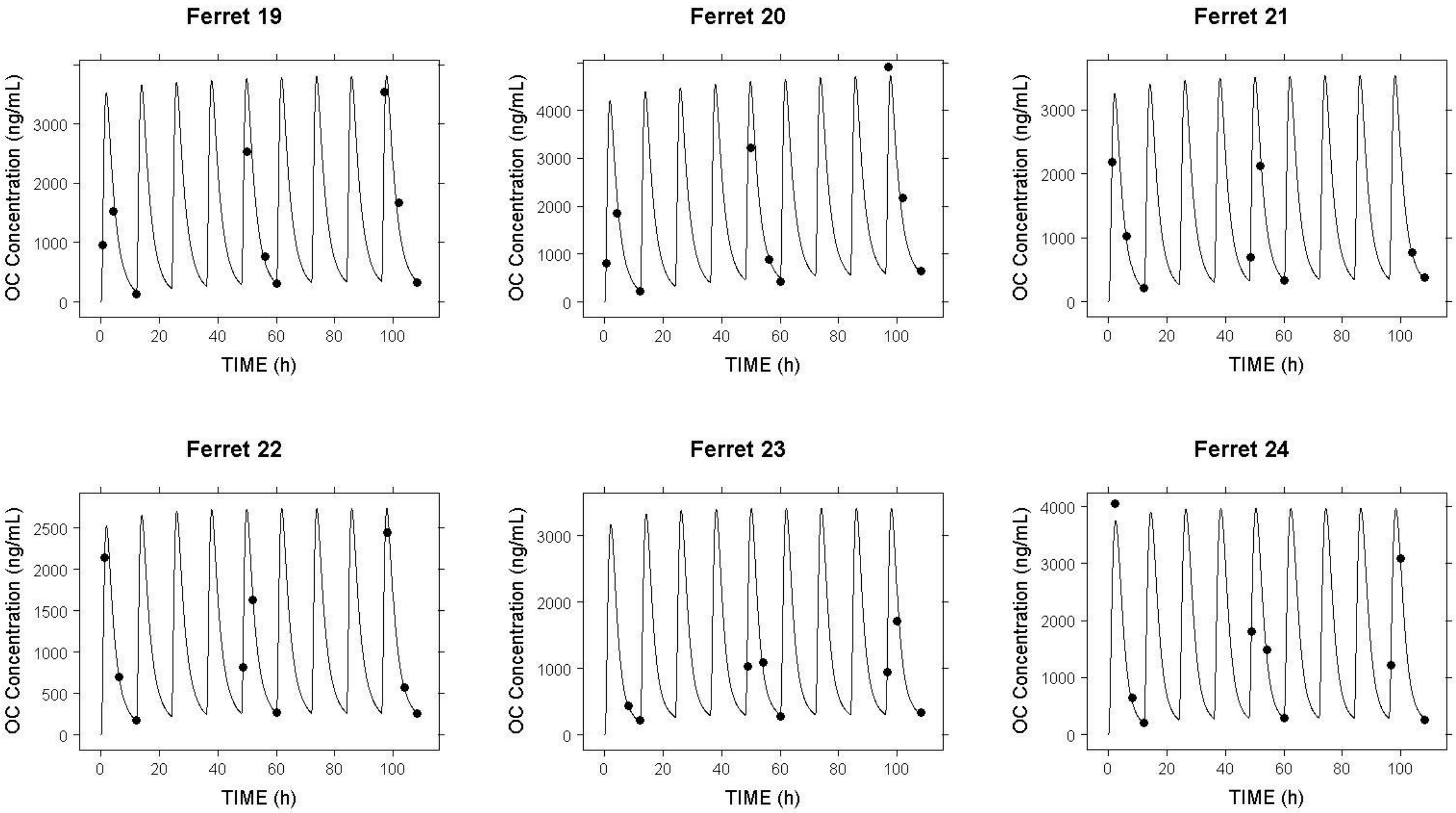
Ferret 19
Ferret 20
Ferret 21
Ferret 22
Ferret 23
Ferret 24
OC Concentration (ng/mL)
TIME (h)
0
20
40
60
80
100
0
1000
2000
3000
4000
500
1500
2500

Study 3: Ferrets were uninfected.

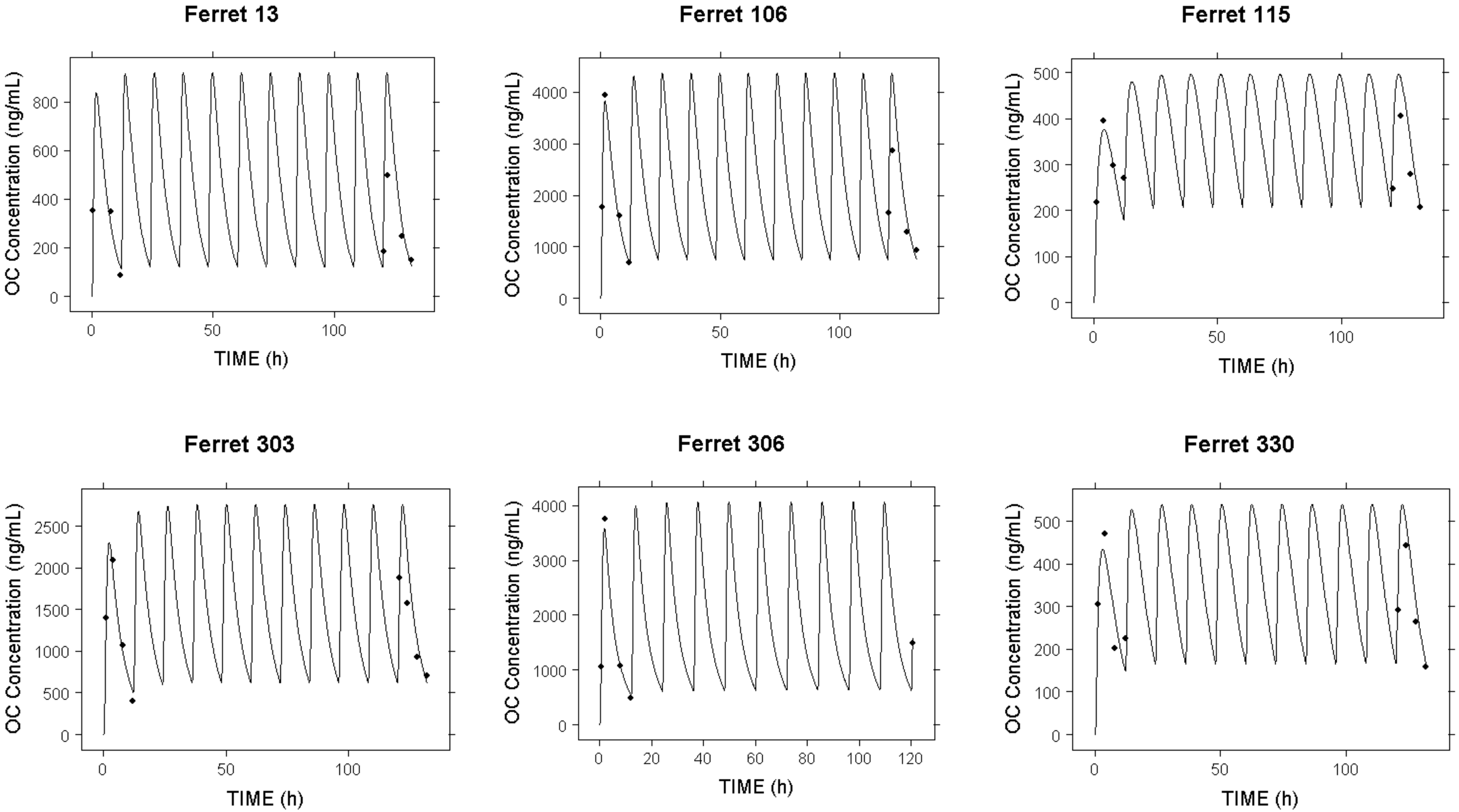

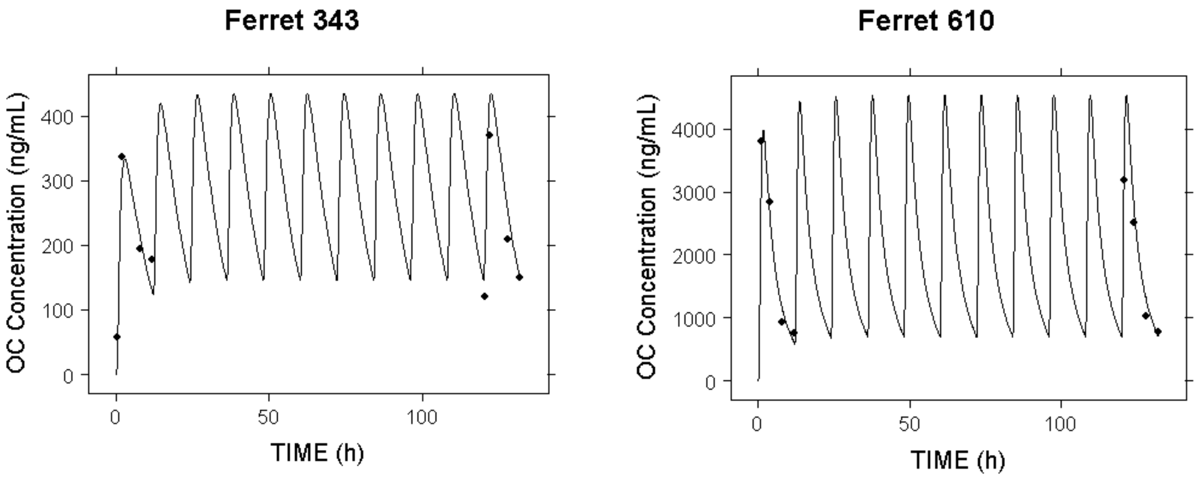

Ferret 343
OC Concentration (ng/mL)
TIME (h)
Ferret 610
OC Concentration (ng/mL)
TIME (h)
